# Taxonomic Demarcation of *Setaria pumila* (Poir.) Roem. & Schult., *S. verticillata* (L.) P. Beauv., and *S. viridis* (L.) P. Beauv. (Cenchrinae, Paniceae, Panicoideae, Poaceae) From Phytolith Signatures

**DOI:** 10.3389/fpls.2018.00864

**Published:** 2018-06-22

**Authors:** Mudassir A. Bhat, Sheikh A. Shakoor, Priya Badgal, Amarjit S. Soodan

**Affiliations:** Plant Systematics and Biodiversity Laboratory, Department of Botanical and Environmental Sciences, Guru Nanak Dev University, Amritsar, India

**Keywords:** grasses, morphotypes, phytoliths, *Setaria* spp., silica, taxonomic demarcation

## Abstract

**Background and Aims:** The role and significance of phytoliths in taxonomic diagnosis of grass species has been well documented with a focus on the types found in foliar epidermis and the synflorescence. The present paper is an attempt to broaden the scope of phytoliths in species diagnosis of grasses by developing phytolith signatures of some species of the foxtail genus *Setaria* P. Beauv. through *in situ* location and physico-chemical analysis of various phytolith morphotypes in different parts of the plant body.

**Methods:** Clearing solution and dry ashing extraction methods were employed for *in situ* location and isolation of phytolith morphotypes respectively. Ultrastructural details were worked out by Scanning Electron Microscopy (SEM) and Transmission Electron Microscopy. Morphometric and frequency data of phytolith morphotypes were also recorded. Biochemical architecture of various phytolith types was worked out through SEM-EDX, XRD, and FTIR analysis. Data were analyzed through Principal Component Analysis and Cluster Analysis.

**Key Results:** *In situ* location of phytoliths revealed species specific epidermal patterns. The presence of cystoliths (calcium oxalate crystals) in the costal regions of adaxial leaf surface of *S. verticillata* (L.) P. Beauv. is the first report for the genus *Setaria*. Our results revealed marked variations in epidermal ornamentation and undulation patterns with a novel “Λ” (Lamda) type of undulated ornamentation reported in *S. verticillata*. Dry ashing method revealed species specific clusters of phytolith morphotypes.

**Conclusions:** The study revealed that phytoliths can play a significant role in resolution of taxonomic identity of three species of *Setaria*. Each species was marked out by a unique assemblage of phytolith morphotypes from various parts of the plant body. Apart from *in situ* location and epidermal patterning, diagnostic shapes, frequency distribution, size dimensions, and biochemical architecture emerged as complementary traits that help in developing robust phytolith signatures for plant species.

## Introduction

The foxtail genus, *Setaria* P. Beauv., so named by the presence of sterile bristles that subtend spikelets in a close panicle, belongs to the “bristle clade” (subtribe Cenchrinae, tribe Paniceae, subfamily Panicoideae) of the grass family Poaceae (Morrone et al., 2012). The genus has a labile morphology requiring additional characters for the resolution of phylogenetic relations among the 113 odd species of the genus (Clayton et al., 2016 onwards). One of the species, the foxtail millet *Setaria italica* (L.) P. Beauv. has been cultivated along with other millets in dryland farming system since prehistoric times (Madella et al., 2016; Weisskopf and Lee, 2016). Some other species of the genus also serve as significant sources of forage and fodder (Aliscioni et al., 2011; Marinoni et al., 2013). Several studies have attempted to resolve the infrageneric (Stapf and Hubbard, 1934; Webster, 1987; Pensiero, 1999) and intergeneric (Webster, 1993, 1995; Veldkamp, 1994; Morrone et al., 2014) relations of the genus. Molecular studies on the chloroplast gene ndhF have revealed polyphyletic nature of the genus with three well supported clades (Kellogg et al., 2009). Even though leaf blade anatomy has traditionally been employed for taxonomic characterization of grasses (Prat, 1936, 1948; Metcalfe, 1960; Ellis, 1979, 1984), the role of anatomical characters in grass taxonomy and phylogeny has been, so to say, rediscovered in the recent past (Ingram, 2010) with *Setaria* P. Beauv. as a model genus (Aliscioni et al., 2016). Apart from epidermal cell patterns and vasculature, phytoliths in leaf epidermis and other parts of the plant body have been utilized for species characterization and taxonomic analysis of grass taxa.

Phytolith studies have been utilized both for characterization of individual *Setaria* species (Rovner, 1971; Hodson et al., 1982) as also for taxonomic demarcation among species within the genus (Zhang et al., 2011; Layton and Kellogg, 2014; Wang et al., 2014; Madella et al., 2016) and from related genera (Hunt et al., 2008; Lu et al., 2009; Out et al., 2014; Wang et al., 2014; García-Granero et al., 2016; Madella et al., 2016). The ever increasing role of phytoliths in the resolution of intrageneric and intergeneric taxonomy of the genus can be ascribed to the simple fact that even among grasses, *Setaria* spp. show exceptional levels of silica accumulation in the form of phytoliths in all parts of the plant body. During the present investigations, an attempt has been made to supplement the information available on the phytolith profiles of three closely related species of the foxtail grass genus through a multiproxy approach and the development of phytolith signatures as additional evidence for their taxonomic demarcation. Analysis of several aspects of phytoliths from different parts of the plant body of the selected species was done through a battery of techniques employed in a logical sequence from *in situ* location of phytolith morphotypes in foliar epidermis to advanced level of physico-chemical analysis involving sophisticated instruments and methodology. In this context, the present study marks a significant advance toward developing a comprehensive and robust framework for the use of data on morphotype diversity, distribution in different parts of the plant body and their ultrastructural and biochemical characterization in identification and taxonomic demarcation of plant taxa.

### Silica and phytolith production in plants

Plants absorb monosilicic acid (H_4_SiO_4_), which is released to the soil by weathering of siliceous minerals, by action of an aquaporin-like channel Low-silicon 1 (*Ls1*) and a proton antiporter Low-silicon 2 (*Ls2*) and polymerizes it into amorphous silica (SiO_2_.nH_2_O) in cell lumens (internal casts), intercellular spaces, and cell walls (external casts) of the parenchymatous tissue (Baker, 1959b; Jones and Handreck, 1967; Rovner, 1971; La Roche, 1977; Bombin, 1984; Piperno, 1988; Mulholland, 1989; Ma et al., 2011; Ma and Yamaji, 2015). A number of unknown silica transporters are believed to be involved in directing silica transfer to different silicification sites (Kumar et al., 2017). Being hard and resistant to dessication and disfiguration, these amorphous silica bodies are commonly called phytoliths [phyton (φ*υτoν*) = plant + lithos (λ*ιθoς*) = stone]. As casts (both internal and external) of plant cells, phytoliths vary in shape, size, frequency, surface ornamentation and other structural features (Ollendorf et al., 1988; Piperno, 1988, 2006; Lu and Liu, 2003; Lu et al., 2009; Zhang et al., 2011; Szabo et al., 2015; Ge et al., 2016). Genetic control of shape, size and frequency of phytoliths has been demonstrated in some monocots (e.g., *Zea mays* L.) and dicots (e.g., *Cucurbita* spp. L.) (Bozarth, 1987; Piperno et al., 2000).

Phytoliths have been implicated in several biological functions including that of providing an endoskeletal framework which prevents wilting (Parry and Smithson, 1958a) and offering resistance to herbivory (Rovner, 1971; Stebbins, 1972, 1981; Coughenour, 1985; Epstein, 1994, 1999), and alleviating biotic (Jones and Handreck, 1967; Gould and Shaw, 1983; Mazumdar, 2011) and abiotic (Hodson et al., 1985; Hodson and Evans, 1995; Lux et al., 2003; Hattori et al., 2005) stress. Phytoliths have also been reported to play a role in checking the rate of transpiration and at the same time reducing the heat load of plants growing in exposed habitats (Jones and Handreck, 1967; Sangster and Parry, 1971; Krishnan et al., 2000).

Ecological functions played by phytoliths include a role in biogeochemical and bio-cycling of silicon in terrestrial ecosystems (Conley, 2002; Gerard et al., 2008; Borrelli et al., 2010; Struyf and Conley, 2012) and sequestration of occluded carbon (Rajendiran et al., 2012; Parr and Sullivan, 2014; Alexandre et al., 2015; Ru et al., 2018; Yang et al., 2018). Isotopic dating of phytolith occluded carbon (PhytOC) has been employed to determine the age of sediments and that of elements of vegetation trapped in these sediments (Parr and Sullivan, 2014). The use of phytoliths in dating of plant fossils can be attributed to the fact that upon death and *in situ* decay of the plant body, phytoliths are released into the soil where they stay through the millenia resisting deformation and destruction by the vagries of geological and climatic conditions. Their long time persistence in the soil make them ideal plant microfossils which have been recovered from sediments as far back as 60 mya in the Cenozoic (Jones, 1964), including the glacials (Twiss et al., 1969; Fredlund et al., 1985) and the Holocene (Baker, 1959a; Crawford, 2009). Phytoliths have been recovered from diverse habitats including swamps (Baker, 1959a), arid zones (Pease and Anderson, 1969), humid areas (Jones and Beavers, 1964) and vegetation types including grasslands and forests (Wilding and Drees, 1973).

Owing to widespread production across several plant groups and excellent preservation as microfossils, phytoliths have found an ever increasing role as proxies in diverse fields of scientific enquiry including archeaobotany of the centers of civilization and cultivation (Schellenberg, 1908; Pearsall, 1978; Rovner, 1983; Piperno, 1984; Shillito, 2013; Gao et al., 2018), paleoecology and paleoclimatology (Rovner, 1971; Carbone, 1977; Fox et al., 1996; Piperno, 2006; Albert et al., 2007), the mapping of ancient land use patterns, and vegetation structure (Gross, 1973; Pearsall and Trimble, 1984; Fisher et al., 1995). Phytolith profiles of present day crop species and soil samples of ancient sites have been compared and calibrated for developing historical calendars for the origin of agriculture and routes of spread and diversification of crop species and calculating the crop ratios (Rovner, 1983; Piperno, 1998, 2009; Pearsall et al., 2003; Albert and Henry, 2004; Fuller et al., 2007; Itzstein-Davey et al., 2007; Tsartsidou et al., 2007; Hunt et al., 2008; Crawford, 2009; Lu et al., 2009; Zhang et al., 2010, 2012; Zhao, 2011; Chen et al., 2012; Madella et al., 2014, 2016; Weisskopf et al., 2014; Out and Madella, 2016; Weisskopf and Lee, 2016), the food and non-food uses of plants in crafts and building materials (Ryan, 2011), agricultural practices (e.g., irrigation, Rosen and Weiner, 1994; Slash-n-burn; Piperno, 1998), paleoagrostology (Piperno and Pearsall, 1998), taphonomy (Madella and Lancelotti, 2012) and colonization of islands and distant lands (Astudillo, 2017).

On account of the wide range of availability and ease of recovery from unused parts of cereals (and other crop species) and the purity of silica obtained, phytoliths have also found a role in nanotechnology (Neethirajan et al., 2009; Qadri et al., 2015). In the contemporary environmental context, phytoliths are being employed as models for assessment of the effects of global warming and climate change (Hongyan et al., 2018).

### Phytoliths in grass systematics

Notwithstanding the above mentioned applications, phytoliths have found the most significant role in taxonomic characterization and demarcation of plant taxa. At this juncture it would be quite instructive to review the landmarks in plant phytolith research that have provided the framework for the use of phytoliths in grass systematics as well. After the revisionary work of (Netolitsky, 1929), attempts were made to identify the marker morphotypes for plant taxa at different levels of taxonomic hierarchy. Within grasses, branched cells were typically associated with *Nardus stricta* L. (Parry and Smithson, 1958a,b). Twiss et al. (1969) expanded the scope of “marker morphotype” approach to major groups within the family through a study of 26 morphotypes of which eight were ascribed to festucoid group, two to chloridoid, and 11 to panicoid grasses and the rest (five) had no particular subfamilial affiliation. Soon afterwards, Rovner (1971) pointed out that a search for “marker” types for plant taxa would run into difficulty on account of “multiplicity” of types within a single species (more so for taxa at higher ranks) and “redundancy” of occurrence of same types “appearing in related as well as taxonomically unrelated species.” Rovner (1971) suggested that assemblages or “type-sets” of phytoliths would provide better taxonomic demarcation among plant species and soil samples.

Apart from types, Mulholland (1989) presented data on frequencies of various types to characterize 19 wild grasses collected from their natural habitats. Piperno and Pearsall (1993) pointed out that phytoliths from reproductive parts proved more useful in separating maize (*Zea mays* L.) from teosinte. This work focused on an organ-specific approach in using phytoliths in taxonomic demarcation of grass species. Pearsall et al. (1995) further narrowed it down to “silicified glumes” as the most revealing in distinguishing cultivated rice (*Oryza sativa* L.) from its wild relatives. Piperno (1998) identified diagnostic morphotypes of phytoliths for the subfamilies Pooideae, Arundinoideae, Chloridoideae, and described the diagnostic and diverse types in the Bambusoideae in great detail. Several subsequent workers have utilized typology and frequency (abundance) approachs to phytolith analysis for taxonomic characterization and demarcation of cultivated and wild grasses (Piperno, 1985; Zhang et al., 2012; Tripathi et al., 2013).

Rudall et al. (2014) employed the shapes of costal phytolith morphotypes and their orientation to elucidate phylogenetic relationships among different grass subfamilies and supported the recognition of three clades within the family. The APP (Anomochloideae, Pharoideae, Puelioideae) clade was treated as the most primitive followed by BEP (Bambusoideae, Ehrhartoideae, Pooideae) and species rich PACCMAD (Panicoideae, Arundinoideae, Chloridoideae, Micrairoideae, Aristidoideae, Danthonioideae) clades. Kealhofer et al. (2015) carried out phytolith analysis of leaf and synflorescence of the foxtail millet [*S. italica* (L.) Beauv.]. In India, Jattisha and Sabu (2015) brought out the taxonomic significance of foliar phytoliths as diagnostic markers in some grasses of South India. More recently, Shakoor et al. (2016) employed phytoliths from underground (root) and aerial (culm, leaf & synflorescence) parts for taxonomic demarcation of two reed grasses, *Arundo donax* L. and *Phragmites karka* (Retz.) Trin. ex Stued.

Parry et al. (1984) marked the biochemical dimension in phytolith characterization by reporting a time dependent accumulation of some elements (K, Cl, P, and S) along with silicon in the silicified microhairs from the lemma of the canary grass, *Phalaris canariensis* L. and giving evidence of genetic control of silicification. In recent years, physico-chemical characterization of phytoliths has been extended to a study of the physical states (as amorphous vs. crystalline), the mineral composition and the study of functional groups and their bonding patterns through sophisticated methods of analysis (Chauhan et al., 2011; Shakoor et al., 2016).

The work reported in this paper is a part of the ongoing program of research on the role of phytoliths in the systematic analysis of grass flora in the area of study. *Setaria* species selected for the present investigations show morphological similarity with one another as well as the foxtail millet *S. italica* (L.) P. Beauv. and are placed closely in keys to species identification of the genus (Layton and Kellogg, 2014). *Setaria viridis* (L.) P. Beauv. had an Asian origin with phylogenetic relations with its domesticated derivative the foxtail millet, *S. italica* with which it remains interfertile (Shi et al., 2008). The second species of the present sample, *S. verticillata* is the polyploid derivative of *S. viridis* (L.) P. Beauv. (Layton and Kellogg, 2014). The third species, *S. pumila* (Poir.) Roem. & Schult. had an African origin (Rominger et al., 2003) but shares a wide distribution with *S. viridis* and growth in mixed populations and is included in the “*S. viridis* clade” of the genus. The foxtail millet, *Setaria italica* would have been a useful and desirable addition to the material but it is not cultivated in the Punjab plains and was thus unavailable for this work. Even though permanent herbarium sheets of this species were available in the departmental herbarium, sufficient material could not have been extracted from them for the present analysis.

## Materials and methods

### Area of study

**Figure 1:**
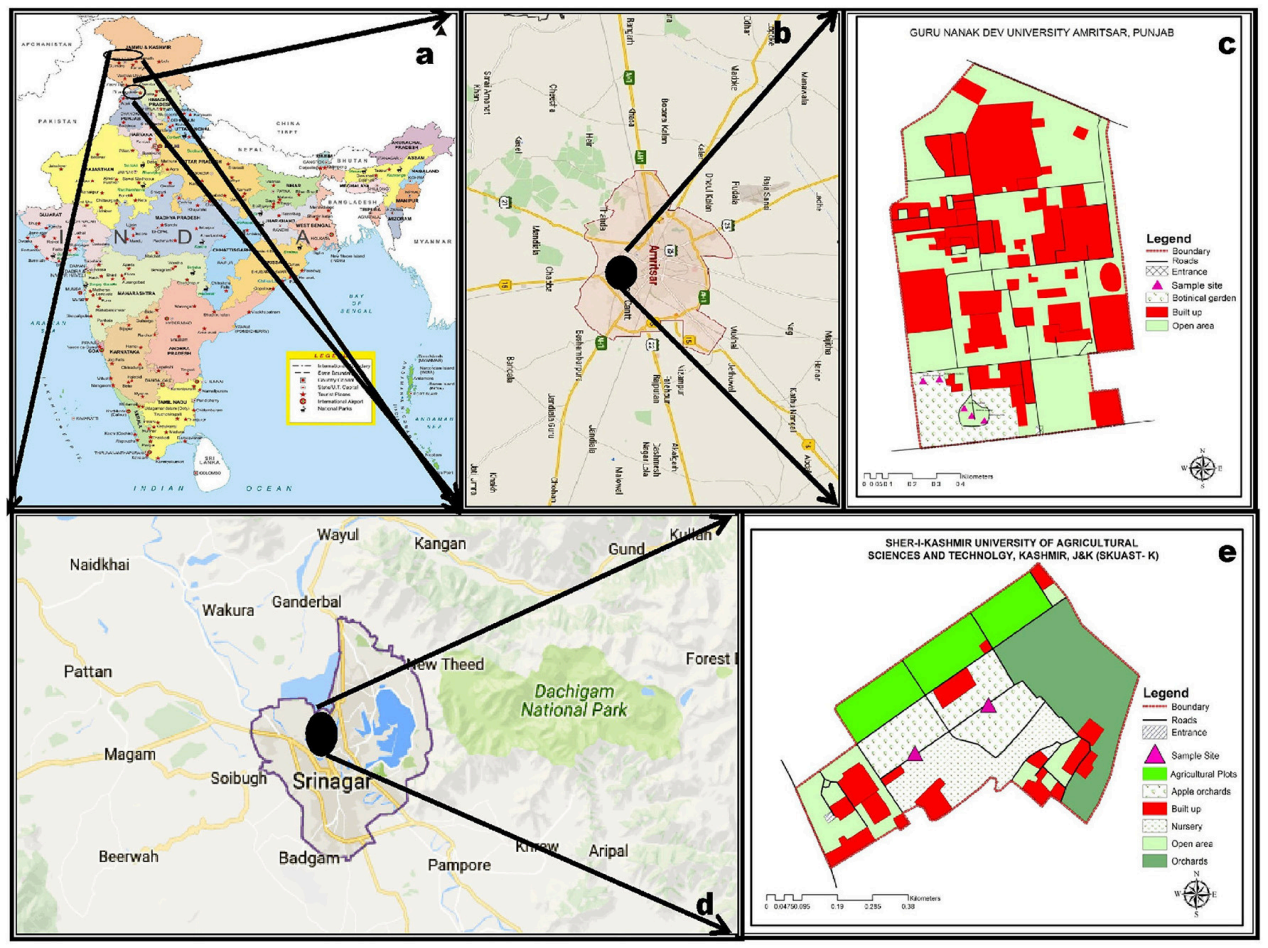
Distribution of sampling sites in India **(a–e)**: *Setaria pumila* (Poir.) Roem. & Schult. and *Setaria verticillata* (L.) P. Beauv. **(b,c)** and *Setaria viridis* (L.) P. Beauv. & Schult. **(d,e)**.

About twenty plant specimens of *S. pumila* and *S. verticillata* were collected from the campus of Guru Nanak Dev University, (32.64 °N and 74.82 °E) Amritsar, Punjab (Figures 1a–c). A similar number of plants of *S. viridis* were collected from the campus of Sher-i-Kashmir University of Agricultural Sciences and Technology, (32.65 °N and 74.81 °E) Srinagar, Jammu & Kashmir (Figures 1d,e). The specimens were collected at flowering and fruiting stage. Taxonomic descriptions and illustrations of the species were made from fresh material in the standard formats of grass description proposed by Grass Phylogeny Working Group (GPWG (Grass Phylogeny Working Group)., 2001) and GPWG (Grass Phylogeny Working Group II). (2011) systems and maintained by the online sources (Clayton et al., 2016; GrassBase—The Online World Grass Flora: The Board of Trustees, Royal Botanic Gardens [online]. Available at http://www.kew.org/data/grasses-db.html and 2. Tropicos (2011) http://www.tropicos.org. Name Search.aspx. 3.eflora of China: http://www.efloras.org. Missouri Botanical Garden, St. Louis, MO and Harvard University Herbaria, Cambridge, MA). The species identity of the specimens was established by comparison of the vegetative and reproductive morphology and micromorphometry with standard descriptions available in works of grass floristics of the world (Bor, 1960; Gould, 1968; Cope, 1982; Gould and Shaw, 1983; Clayton and Renvoize, 1986; Watson and Dallwitz, 1992; Kellogg, 2015; Soreng et al., 2017 and the region Sharma and Khosla, 1989; Kumar, 2014). For preparation of herbarium sheets, three dried specimens for each of the species have been deposited in the Herbarium of the Department of Botanical and Environmental Sciences, Guru Nanak Dev University, Amritsar (Voucher nos. 7373 to 7381).

### Phytolith analysis

About five to ten plants remaining intact after taxonomic descriptions and dry preservation for hebarium specimens, were dismembered into underground (root) and above ground (culm, leaf and synflorescence) parts. The material was homogenized (part wise) into lots. Some of the material from each lot was preserved in 70% ethanol at 4°C for *in situ* location of phytoliths. The rest of the material in each lot was washed to clear dust and adhering soil particles, sundried and stored in plastic jars for dry ashing and further analysis.

Methodology of the present study followed a logical and systematic sequence from *in situ* visualization of the phytoliths in the leaf epidermis to dry ashing of plant parts for disarticulation of individual morphotypes for recording qualitative (morphotypic) data and collection of quantitative (micromorphometric) data on phytoliths among the species and their parts. Quantitative assessment also included frequency distribution of various morphotypes. After data collection at the level of light microscopy (LM), scanning electron microscopy (SEM) of morphotypes was carried out to record their surface ornamentations and three dimensional structures. Transmission electron microscopy (TEM) was employed to study variations in texture, interplanar spacing, and crystallinity of various morphotypes. EDX analysis was employed to study elemental composition of phytolith morphotypes and the rhizospheric soil. XRD analysis revealed the physical phases of silica and other minerals in the phytoliths. Similarly, FTIR analysis was carried out to know the functional groups of phytoliths from different plant parts.

### *In situ* location

A study of *in situ* location and epidermal patterning of phytoliths on both adaxial and abaxial surfaces of the leaf was carried out according to the method of Clarke (1959) with some modifications. The leaf segments from mature leaves were boiled in tubes for 5–10 min in distilled water. After cooling down the tubes, leaf segments were put in ethanol (absolute) and heated gently (80°C) in a water bath till they were decolorized. Thereafter, the segments were immersed in a solution of lactic acid and chloral hydrate (3:1 v/v) and boiled again for 20–30 min in a water bath. After clearing, they were placed on clean ceramic tiles with the adaxial surface upwards and the epidermis was peeled off the middle part of mature leaf blades. Similarly, peelings from abaxial surface of leaf segments were obtained. Epidermal peelings were stained in Gentian Violet and passed through a dehydration series of ethanol (30% through 50, 70, 90% and absolute ethanol) and mounted in DPX for light microscopy and microphotography with a Micro Image Projection System (MIPS-USB 0262) mounted on an Olympus Binocular and connected to a computer for imaging.

### Dry ashing method

The standard protocol of Carnelli et al. (2001) with some modifications was employed for dry ashing of the plant material. The dried and stored material of individual parts was taken from the plastic jars, further dried in an oven, weighed and transferred to porcelain crucibles. The material was incinerated at 550°C for 4–6 h to ashes. The crucibles were taken out of the furnace, allowed to cool and ash contents were transferred to test tubes. A sufficient amount of hydrogen peroxide (30%) was added to submerge the ash and the test tubes were kept at 80°C for 1 h in a water bath. The mixture was decanted and rinsed twice in distilled water. Hydrochloric acid (10%) was added to the pellet and incubated at 80°C for 1 h. Thereafter, the mixture was washed in distilled water and centrifuged for 15 min at 7,500 rpm. The supernatant was decanted off and the pellet was washed twice in distilled water. The process was repeated till the pellet was clear. Finally, the pellet was oven dried for 24 h at 60°C to a powder form and weighed. The silica content (%) was calculated by the formula: final ash content/dry mass × 100.

A small amount (ca. 0.1 mg) of dried ash was dipped in 10 ml of Gentian Violet in a watch glass and stirred. A drop of this mixture was put on a glass slide and covered with a cover slip. The slides were heated gently and excess stain drained off with a filter paper. Ten slides were prepared for each sample. Morphotypes of phytoliths were photographed by Olympus Micro Image Projection System (MIPS-USB 0262) at a uniform magnification (40X). The phytoliths isolated by the dry ashing method from underground (root) and aerial (culm, leaf, and synflorescence) parts showed considerable diversity of phytolith morphotypes in terms of their shapes and were classified according to the International Code of Phytolith Nomenclature (ICPN 1.0; Madella et al., 2005). Some of the morphotypes whose description was not available in the ICPN nomenclature were classified as per the schemes presented in Table 1.

**Table 1:** Description of different phytolith morphotypes and their diagnostic potential in *Setaria* spp. of the present study.

**S.No**	**Phytolith Morphotypes**	**Acronym**	**Morphotype description (as per ICPN 1.0)**			***Setaria pumila*** **(Poir.) Roem. & Schult.[SP]**				***Setaria verticillata*** **(L.) P. Beauv. [SVC]**				***Setaria viridis*** **(L.) P. Beauv. [SV]**				**Diagnostic for**	**Ubiquity**	**Reference (s)**
			**First descriptor**	**Second descriptor**	**Third descriptor**															
						**Rt**	**Cl**	**Lf**	**Synflo**	**Rt**	**Cl**	**Lf**	**Synflo**	**Rt**	**Cl**	**Lf**	**Synflo**			
1.	Acicular	ACL	Acicular	—————	—————	–	–	–	–	–	–	–	+	–	–	–	+	SVC & SV	0.66 (0.17)	Madella et al., 2005
2.	Bilobate class I	BCI	Bilobate class I	—————	—————	+	–	–	+	–	–	–	–	–	–	–	–	SP	0.33 (0.17)	Gallego and Distel, 2004
3.	Bilobate class II	BCII	Bilobate class II	—————	—————	–	–	–	–	–	–	–	–	–	–	+	+	SV	0.33 (0.17)	Gallego and Distel, 2004
4.	Bilobate class III	BCIII	Bilobate class III	—————	—————	–	–	+	–	–	–	+	–	–	–	–	–	SP & SVC	0.66 (0.17)	Gallego and Distel, 2004
5.	Bilobate class IV	BCIV	Bilobate class IV	—————	—————	–	–	–	–	–	–	+	–	–	–	+	–	SVC & SV	0.66 (0.17)	Gallego and Distel, 2004
6.	Bilobate class V	BCV	Bilobate class V	—————	—————	+	–	+	–	+	–	–	–	–	–	+	–	—–	0.66 (0.33)	Gallego and Distel, 2004
7.	Bilobate class VI	BCVI	Bilobate class VI	—————	—————	+	–	+	–	–	–	–	–	–	–	+	–	SP & SV	0.66 (0.25)	Gallego and Distel, 2004
8.	Bilobate class VII	BCVII	Bilobate class VII	—————	—————	–	–	–	–	–	–	+	–	–	–	+	–	SVC & SV	0.66 (0.17)	Gallego and Distel, 2004
9.	Bilobate class VIII	BCVIII	Bilobate class VIII	—————	—————	–	–	–	–	–	–	–	–	–	–	+	–	SV	0.33 (0.08)	Blinnikov, 2005
10.	Blocky crenate	BC	Blocky	Crenate	—————	–	–	–	–	–	+	–	–	–	–	–	–	SVC	0.33 (0.08)	Barboni et al., 2010
11.	Blocky irregular	BIR	Blocky	Irregular	—————	+	–	+	+	+	+	+	+	+	+	+	+	—–	1.0 (0.91)	Barboni et al., 2010
12.	Blocky polyhedral	BPH	Blocky	Polyhedral	—————	–	+	+	+	+	+	+	–	+	+	+	–	—–	1.0 (0.75)	Blinnikov, 2005
13.	Carinate	CRN	Carinate	—————	—————	–	–	–	–	–	–	–	–	–	+	–	+	SV	0.33 (0.17)	Madella et al., 2005
14.	Clavate	CLV	Clavate	—————	—————	–	–	+	+	–	–	+	–	–	+	+	–	—–	1.0 (0.40)	Madella et al., 2005
15.	Columellate elongate	CE	Columellate	Elongates		–	–	–	+	–	–	–	+	–	–	–	–	SP & SVC	0.66 (0.17)	Madella et al., 2005
16.	Crescent moon	CM	Crescent moon	—————	—————	–	–	–	–	+	–	–	–	–	–	–	–	SVC	0.33 (0.08)	Fernandez Honaine et al., 2006
17.	Cross	CRS	Cross	—————	—————	–	–	+	–	–	–	+	+	–	–	–	–	SP & SVC	0.66 (0.25)	Madella et al., 2005
18.	Cuboid	CUB	Cuboid	—————	—————	–	–	+	–	+	+	–	–	+	+	+	–	—–	1.0 (0.50)	Ellis, 1979
19.	Cuneiform bulliform	CB	Cuneiform	—————	Bulliform	+	+	–	+	+	–	+	+	–	+	–	+	—–	1.0 (0.66)	Madella et al., 2005
20.	Cylindrical	CYD	Cylindrical		—————	–	–	+	–	+	–	–	–	–	–	–	+	—–	1.0 (0.25)	Madella et al., 2005
21.	Echinate elongate	EE	Elongate	Echinate	—————	–	+	–	+	–	–	–	+	–	–	+	–	—–	1.0 (0.25)	Madella et al., 2005
22.	Elongate irregular	EIR	Elongate	Irregular	—————	–	–	+	+	–	+	+	–	–	+	–	–	—–	1.0 (0.41)	Gallego and Distel, 2004
23.	Elongate with concave ends	ECE	Elongate	Concave ends	—————	–	–	–	–	+	–	–	–	–	–	–	–	SVC	0.33 (0.08)	Gallego and Distel, 2004
24.	Epidermal element	EPE	—————	—————	Epidermal element	–	–	–	+	–	–	–	–	–	–	–	+	SP & SV	0.66 (0.17)	Gallego and Distel, 2004
25.	Epidermal element with columellate extensions	EECE	—————	Columellate extensions	Epidermal element	–	–	–	–	–	–	–	+	–	–	–	–	SVC	0.33 (0.08)	Madella et al., 2005
26.	Epidermal element with short silica cells and stomata	EESSCS	—————	—————	Epidermal element with short silica cells and stomata	–	–	–	–	–	–	–	–	–	–	–	+	SV	0.33 (0.17)	Madella et al., 2005
27.	Epidermal element with undulated ridges	EEUR	—————	Undulated ridges	Epidermal element	–	–	–	+	–	–	–	–	–	+	–	–	SP & SV	.66 (0.17)	Gallego and Distel, 2004
28.	Epidermal papillate	EP	—————	Papillate	Epidermal	–	–	–	–	–	–	–	–	–	–	–	+	SV	0.33 (0.08)	Gallego and Distel, 2004
29.	Facetate elongate	FE	Elongate	Facetate	—————	–	–	–	+	–	–	–	–	–	–	–	–	SP	0.33 (0.08)	Madella et al., 2005
30.	Globular echinate	GE	Globular	Echinate	—————	–	–	–	–	+	–	–	–	–	+	+	–	SVC & SV	0.66 (0.25)	Madella et al., 2005
31	Globular granulate	GG	Globular	Granulate	—————	+	–	+	+	–	–	+	–	+	–	+	–	—–	1.0 (0.50)	Madella et al., 2005
32.	Globular polyhedral	GPH	Globular	Polyhedral	—————	+	+	+	+	+	–	+	+	–	+	+	+	—–	1.0 (0.83)	Tsartsidou et al., 2007
33.	Globular psilate	GPI	Globular	Psilate	—————	–	–	–	–	+	+	–	–	+	+	–	+	SVC & SV	0.66 (0.41)	Madella et al., 2005
34.	Half moon	HM	Half moon	—————	—————	–	–	–	–	–	+	–	–	–	–	–	–	SVC	0.33 (0.08)	Morris et al., 2009
35.	Horned tower	HT	Horned tower	—————	—————	–	–	–	+	–	–	+	–	–	–	–	–	SP & SVC	0.66 (0.17)	Twiss et al., 1969
36.	Macrohairs	MH	—————	—————	Macrohairs	–	–	–	+	–	–	+	–	–	–	+	–	—–	1.00 (0.25)	Madella et al., 2005
37.	Nodular bilobate	NBL	Nodular bilobate	—————	—————	+	–	+	–	–	–	+	–	–	–	+	–	—–	1.0 (0.33)	Fahmy, 2008
38.	Oblong	OBL	Oblong	—————	—————	+	–	–	–	–	–	–	–	+	–	–	+	SP & SVC	0.66 (0.25)	Madella et al., 2005
39.	Ovate	OVT	Ovate	—————	—————	–	–	+	–	–	+	_	–	–	–	+	–	—–	1.0 (0.25)	Madella et al., 2005
40.	Parallelepipedal bulliform cell	PBFC	Parallelepipedal	—————	Bulliform cell	–	–	+	–	+	–	–	–	+	–	–	+	—–	1.0 (0.33)	Madella et al., 2005
41.	Plates	PLT	Plates	—————	—————	–	–	–	+	–	–	–	–	–	+	+	–	SP & SV	0.66 (0.25)	Blinnikov, 2005
42.	Polylobate	PL	Polylobate	—————	—————	+	–	–	–	–	–	–	–	–	–	–	–	SP	0.33 (0.08)	Fahmy, 2008
43.	Polylobate irregular	PLIR	Polylobate	Irregular	—————	–	–	–	–	–	–	–	+	–	–	–	–	SVC	0.33 (0.08)	Fahmy, 2008
44.	Prickle hair	PH	—————	—————	Prickle hair	–	–	+	+	–	–	+	–	–	–	+	–	—–	1.0 (0.33)	Madella et al., 2005
45.	Prickly elongate	PE	Elongate	Prickly	—————	–	–	–	–	–	–	–	–	–	–	–	+	SV	0.33 (0.08)	Ellis, 1979
46.	Rectangular	RT	Rectangular	—————	—————	+	–	+	–	+	–	+	+	+	+	–	–	—–	1.0 (0.58)	Madella et al., 2005
47.	Rondel	RD	Rondel	—————	—————	–	–	–	–	–	–	–	+	–	+	–	–	SVC & SV	0.66 (0.17)	Madella et al., 2005
48.	Scutiform	STF	Scutiform	—————	—————	+	–	+	–	–	+	+	+	+	–	+	+	—–	1.0 (0.66)	Madella et al., 2005
49.	Sinuate elongate	SnE	Elongate	Sinuate	—————	–	+	–	+	–	+	–	–	–	–	+	–	—–	1.0 (0.33)	Madella et al., 2005
50.	Sinuate elongate with convex ends	SnECE	—————	Sinuate with convex ends	—————	–	+	–	–	–	–	–	–	–	–	–	–	SP	0.33 (0.08)	Gallego and Distel, 2004
51.	Smooth elongate	SmE	Elongate	Smooth	—————	–	+	+	+	+	+	–	+	–	+	+	+	—–	1.0 (0.75)	Madella et al., 2005
52.	Stomata	STM	—————	—————	Stomata	–	–	–	+	–	–	–	–	–	–	–	–	SV	0.33 (0.08)	Gallego and Distel, 2004
53.	Tabular simple	TBS	Simple tabular	—————	—————	–	–	+	–	+	–	–	–	–	–	–	–	SP & SVC	0.66 (0.17)	Madella et al., 2005
54.	Tabular irregular	TIR	Tabular	Irregular	—————	+	+	–	–	–	–	+	–	–	–	+	–	—–	1.0 (0.33)	Barboni et al., 2010
55.	Tabular polyhedral	TPH	Tabular	Polyhedral	—————	–	–	–	–	–	–	–	–	–	+	+	–	SV	0.33 (0.17)	Barboni et al., 2010
56.	Tracheid	TRCHD	—————	—————	Tracheid	–	–	–	+	–	–	–	–	–	–	–	–	SP	0.33 (0.08)	Madella et al., 2005
57.	Trapezoid	TZ	Trapezoid	—————	—————	+	+	+	+	+	+	+	+	+	+	+	+	—–	1.0 (1.00)	Madella et al., 2005
58.	Triangular	TRN	Triangular	—————	—————	–	–	–	–	+	–	–	–	+	+	–	+	SVC & SV	0.66 (0.33)	Gallego and Distel, 2004

*Rt, Root; Cl. Culm; Lf, Leaf; Syn, Synflorescence; (+), Presence; (–), Absence of particular phytolith morphotype; SP, Setaria pumila (Poir.) Roem. & Schult.; SVC, Setaria verticillata (L.) P. Beauv.; SV, Setaria viridis (L.) P. Beauv*.

### Morphometry

Morphometric measurements of phytolith morphotypes were made using Image J software (version 1.46r.). A total of 5 morphometric parameters of size and shape descriptors were recorded on a minimum sample size calculated as per recommendations of the International Committee for Phytolith Morphometrics (ICPM, Ball et al., 2016) by the formula:

$$\begin{array}{l} N_{\text{min}}=Z_{\text{α}/2}^{2}\times S^{2}/{\left(ME\right)}^{2} \end{array}$$

Where: **N**_min_ = the minimum adequate sample size; ${\text{Z}}_{\text{α}/2}^{2}$ = 1.64, which is the square of the two tailed Z value for level of significance (α) = 0.10; **S**^2^ = the variance, and **(ME)**^2^ = the square of the permissible margin of error (in this case 0.05) × the sample mean. This calculation estimates the minimum adequate sample size required for 95% confidence that the sample means are within 5% deviation from actual population means.

### Scanning electron microscopy (SEM)

For SEM, dry ash was spread evenly over the stubs with the help of double-sided adhesive tape under the stereoscope. Silver paint was applied on edges of the stub and the samples dried overnight at 40°C. The next day, stubs were coated with graphite using a vacuum evaporator and later coated with gold by a sputter coater (QUORUM) and imaged under SEM (CARL ZEISS EVO 40) at an accelerating voltage of 40 KV.

### Transmission electron microscopy

TEM micrographs were obtained using a JEOL JEM-2100 operating at 200 keV. Samples were prepared by suspending a small quantity of powder (crushed in pestle and motar) in double distilled water (DDW). The samples were sonicated for 30 min and a drop of material was placed on a carbon coated copper grid. The grids were dried on filter paper using an electric lamp and were subsequently analyzed. Structural details as well as the chemistry of the samples were worked out by High Resolution Transmission Electron Microscopy (HRTEM) and Selected Area Electron Diffraction (SAED) of various phytolith types.

### Biochemical architecture

Elemental analysis of phytolith morphotypes and soil samples were carried out with Scanning Electron Microscope-Energy Dispersive X-ray analysis (SEM-EDX). Infrared spectra of silica powder were obtained on a Fourier Transform Infrared (FTIR) Spectrophotometer (System92035, Perkin-Elmer, England) at room temperature using the standard KBr method. The functional group spectra were recorded over a wavelength range of 500–4,000 cm^−1^. X-ray Diffraction (XRD) studies were performed on powder XRD system (Bruker D8 Advance) using Cu Kα radiation (k = 1.5418 Å) in the 2θ (Bragg's angle) at a range of 20–70. The data were analyzed for presence of different polymorphic structures of silica and other compounds using the origin pro 8 software and following the notation of the Joint Committee on Powder Diffraction.

Elemental composition of rhizospheric soil samples was carried out with SEM-EDX. Soil samples (ca. 5 g) from the rhizospheric region of the specimens taken for phytolith analysis were collected and ground into fine powder. Small bits of the powder were spread uniformly on the stubs and were scanned using Energy Dispersive X-ray analyzer coupled with the SEM through *Inca software*.

### Statistical analysis

Descriptive statistics of morphometric and elemental composition data was carried out with the help of paleontological statistics (PAST) software (Hammer et al., 2001). Cluster analysis of presence/absence data of bilobate classes of phytoliths and Principal component analysis (PCA) of morphometric and elemental composition data was carried out using C2 data analysis software (Juggins, 2003). This software has also been used for plotting the stratigraphic diagram of the frequency data of phytoliths. Pearson's coefficient of association of phytolith morphotypes of the three species were also calculated employing computer programs developed for the purpose.

## Results and discussion

Taxonomic descriptions of grasses include several (vegetative and reproductive) characteristics that help to characterize and classify taxa from subfamily down to the species and infra specific levels. Morphological and morphometrical characters that diagnose *Setaria pumila, S. verticillata*, and *S. viridis* from one another are presented in Supplementary Table 1. Whereas qualitative characteristics provide a clear cut account of similarities and dissimilarities in paired comparisons, quantitative characteristics show overlapping ranges and cryptic distinctions requiring additional evidence for taxonomic resolution. In the present context, phytolith analysis was employed to supplement and substantiate taxonomic demarcation among the three species of the genus *Setaria* P. Beauv.

### Epidermal patterns

Leaf epidermal characteristics play an important role in taxonomic demarcation of grass taxa due to the unique arrangement of epidermal long and short cells in the costal and intercostal regions (Prat, 1936, 1948; Metcalfe, 1960; Ellis, 1979; Hilu, 1984; Rudall et al., 2014).

The present study has revealed two distinct distribution patterns of silica cells and associated epidermal cells. The first one comprises long-short cell alternation in the intercostal region of the epidermis and the second one includes axial rows of closely spaced short silica cells in the costal region. These costal rows of silica bodies are separated from each other by a single short intervening cell known as the cork cell and are considered highly diagnostic in grasses (Prasad et al., 2011). The intervening cells are relatively thin walled, but resemble silica bodies in size and shape.

The underlying causes for the concentration of the silica bodies over the veins remain unknown though there is an apparent positive correlation between silica deposition and proximity to lignified tissues of the vascular bundles. Indirect support for this association between lignin and silica deposition comes from studies indicating a correlation between silica deposition and lignin metabolism in grasses (Schoelynck et al., 2010).

**Figure 2:**
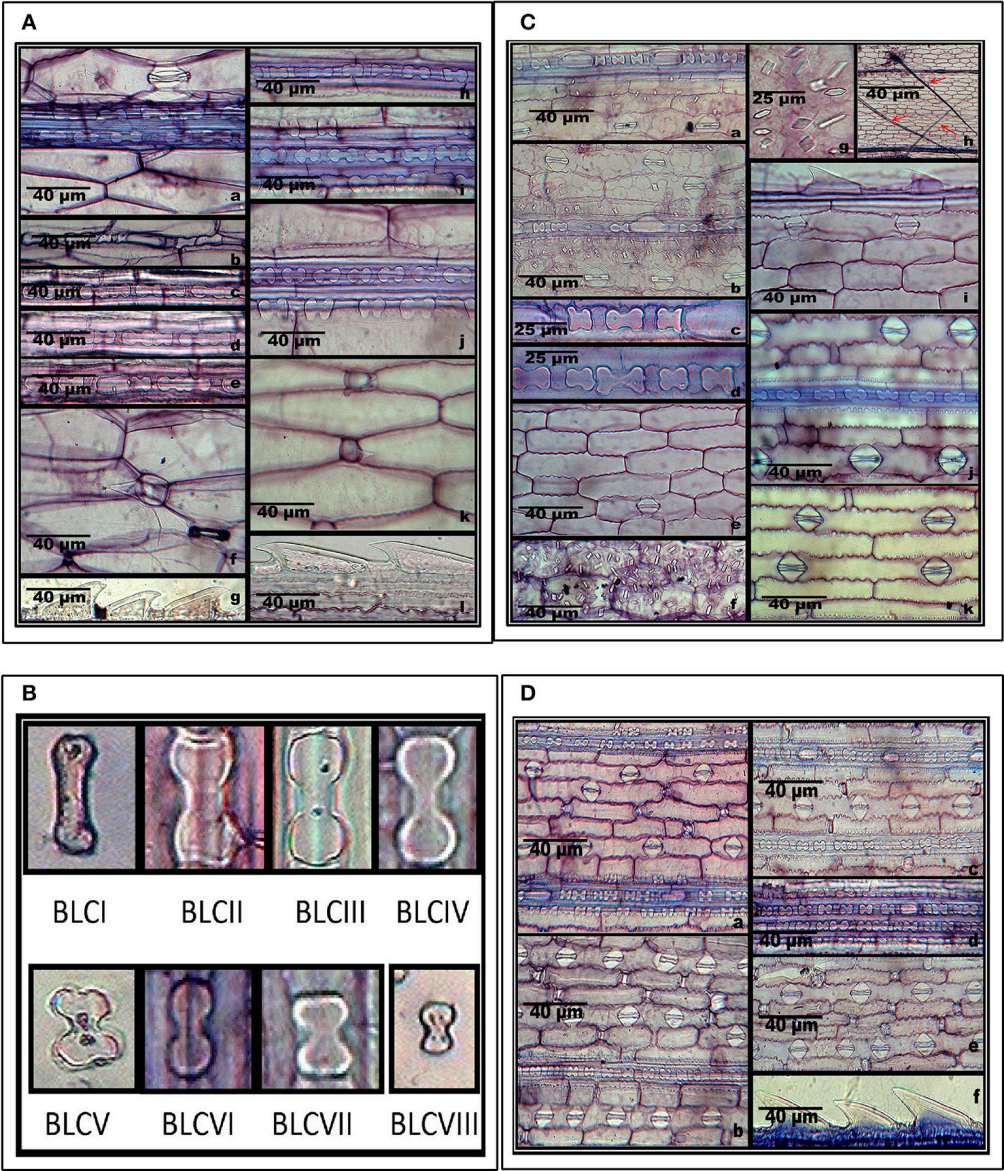
**(A)** *In-situ* location of phytoliths in leaf epidermis of *Setaria pumila* (Poir.) Roem. & Schult. Adaxial surface **(a–g)** and abaxial surface **(h–l). (B)** Morphological classification of bilobate phytolith morphotypes (data in Supplementary Table 4). **(C)** *In-situ* location of phytoliths in leaf epidermis of *Setaria verticillata* (L.) P. Beauv. Adaxial surface **(a–i)** and abaxial surface **(j–k). (D)** *In-situ* location of phytoliths in leaf epidermis of *Setaria viridis* (L.) P. Beauv. & Schult. Adaxial surface **(a,b)** and abaxial surface **(c–f)**.

Supplementary Table 2 summarizes epidermal patterning and the distribution of silica cells and other associated epidermal cells on the adaxial and abaxial leaf surfaces of grass species under investigation. *S. pumila* revealed one to three axially oriented rows of bilobate phytoliths with each bilobate phytolith flanked by silica cork cell in the coastal region on the adaxial surface of cleared leaf segments (Figures 2Aa–e). It also showed the presence of nodular bilobate phytoliths (Figure 2Aa). The costal rows of silica cells showed the presence of prickle hairs (Figure 2Ae). The intercostal region on adaxial surfaces completely lacked silica cells except for occasional prickle hairs (Figure 2Af) with those on the margin having the length of base greater than the barb (Figure 2Ag). The abaxial surface of cleared leaf segments of *S. pumila* presented a different scenario. The costal region showed 1–3 bilobate to cross shaped silica cells with each bilobate/cross pair of silica cells separated by silica cork cells (Figures 2Ah–j). The intercostal region of the abaxial surface in *S. pumila* showed prickle hairs between each pair of epidermal long cells in alternating axial rows (Figures 2Ak,l). The margins on abaxial surfaces showed prickle hairs with a much higher base to barb length ratio than those on the margins of adaxial surfaces.

We have classified bilobate phytolith morphotypes into eight subtypes based upon the length of their shank (the interconnecting segment between two lobes) and the shape of the outer margin of their lobes as proposed by Lu and Liu (2003) (Supplementary Tables 2, 3 and Figure 2B). The bilobate shape is highly conserved and has been employed in identification of grass species (Lu and Liu, 2003; Gallego and Distel, 2004; Fahmy, 2008). The costal region on adaxial surface of *S. pumila* showed three structural variants of the bilobate phytoliths, (III, V, and VI) out of a total eight variants of bilobates recorded from different parts of *Setaria* species (Supplementary Table 3 and Figure 2B). The bilobate and nodular bilobate type of phytoliths with each lobate pair separated by silica cork cells have been reported in the costal region of *S. pumila* (Sharma and Kaur, 1983; Ishtiaq et al., 2001; Shaheen et al., 2011). However, these authors did not report structural variations within the bilobates as recorded in the present investigations.

*S. verticillata* showed an axial row of phytoliths comprising of 3–6 bilobates, a cross and a nodular bilobate flanked by prickle hairs, with each phytolith pair separated by silica cork cells in the costal region (Figures 2Ca–d). The costal region on adaxial surfaces of *S. verticillata* had only two structural variants of bilobate phytoliths (VII and IV as compared to three variants in *S. pumila* (Supplementary Table 3). Shaheen et al. (2011) reported bilobates on adaxial surfaces of the costal region of *S. verticillata*. However, this work made no mention of the presence of the nodular bilobate type of phytolith in the costal region on adaxial surfaces. The intercostal regions lacked silica cells and prickle hairs but showed the presence of long hairs (Figures 2Ce,h) in contrast to *S. pumila* in which prickle hairs were present and long hairs were completely absent. The presence of cystoliths (calcium oxalate crystals) on the adaxial epidermal surface of *S. verticillata* leaf as quadrihedrons and hexahedrons has emerged as the most important diagnostic feature of the species. The cystoliths showed greater concentration in costal rather than the intercostal regions (Figures 2Cf,g). Even though cystoliths have been reported from leaf epidermis in several other grass species (Benecke, 1903; Sato, 1968; Dayanandan et al., 1977; Sato and Shibata, 1981; Lerseten, 1983; Prasad et al., 2005), the present study is the first report of cystoliths for the genus *Setaria*. The abaxial surface in the costal regions showed a single axial row of bilobate and nodular bilobate types of phytoliths (Figure 2Cj). The bilobate class revealed two structural variants (III and IV). The intercostal region showed 1–2 stomatal files of high domed stomata (Figure 2Ck). The margins on abaxial surface showed the presence of prickle hairs with base lengths greater than the barb (Figure 2Ci).

*S. viridis* showed, on the adaxial surfaces 1-4 axial rows of bilobate and nodular bilobate type of phytoliths in the costal region with each phytolith pair flanked by silica cork cells (Figures 2Da,b). Three variants of bilobate phytoliths were present in *S. viridis* (II, IV, and V). These phytoliths are flanked by a pair of prickle hairs in the costal region. The intercostal region showed prickle hairs between each epidermal long cell pair. In contrast to *S. pumila* and *S. verticillata*, the intercostal regions of *S. viridis* occasionally showed a single row of phytoliths. *S. viridis* abaxial leaf surface had 1–3 rows of bilobate phytoliths with occasional nodular bilobate types in the costal region (Figures 2Dc,d). The bilobate class included two structural variants (V and VI). The species had small one celled prickle hairs in the intercostal regions in addition to prickle hairs with bases smaller than the barb on the leaf margins (Figures 2De,f).

### Epidermal ornamentation and undulation patterns

The ornamentation and undulation patterns of epidermal long cells of synfloresence bracts have been put into three categories *viz.*, Ω-undulated, η-undulated, and n-undulated ornamentations (Lu et al., 2009). We propose another undulated ornamentation which can be represented by the symbol ‘Λ’ (Greek-Lamda) and further categorize it into three subtypes: Λ- I, Λ- II, and Λ- III. The Λ-type of undulations were classified on the width of the base and the length of the lateral extensions. If the width of the base and its length was nearly equal, it was put as Λ-I type and if the length was three times the base of lateral extensions, it was recognized as Λ-II type. Similarly, if the length of the lateral extension is more than thrice the width of the base of the extension, it was put as Λ-III. The Ω and η-undulated ornamentations are generally present on the lemmas and palea and have been further put into subtypes based on the degree of undulations as Ω-I, Ω -II & Ω-III and η-I, η-II, η-III respectively (Lu et al., 2009). n-undulated ornamentations were reported on the margins of lemmas and paleas (Zhang et al., 2011).

**Figure 3:**
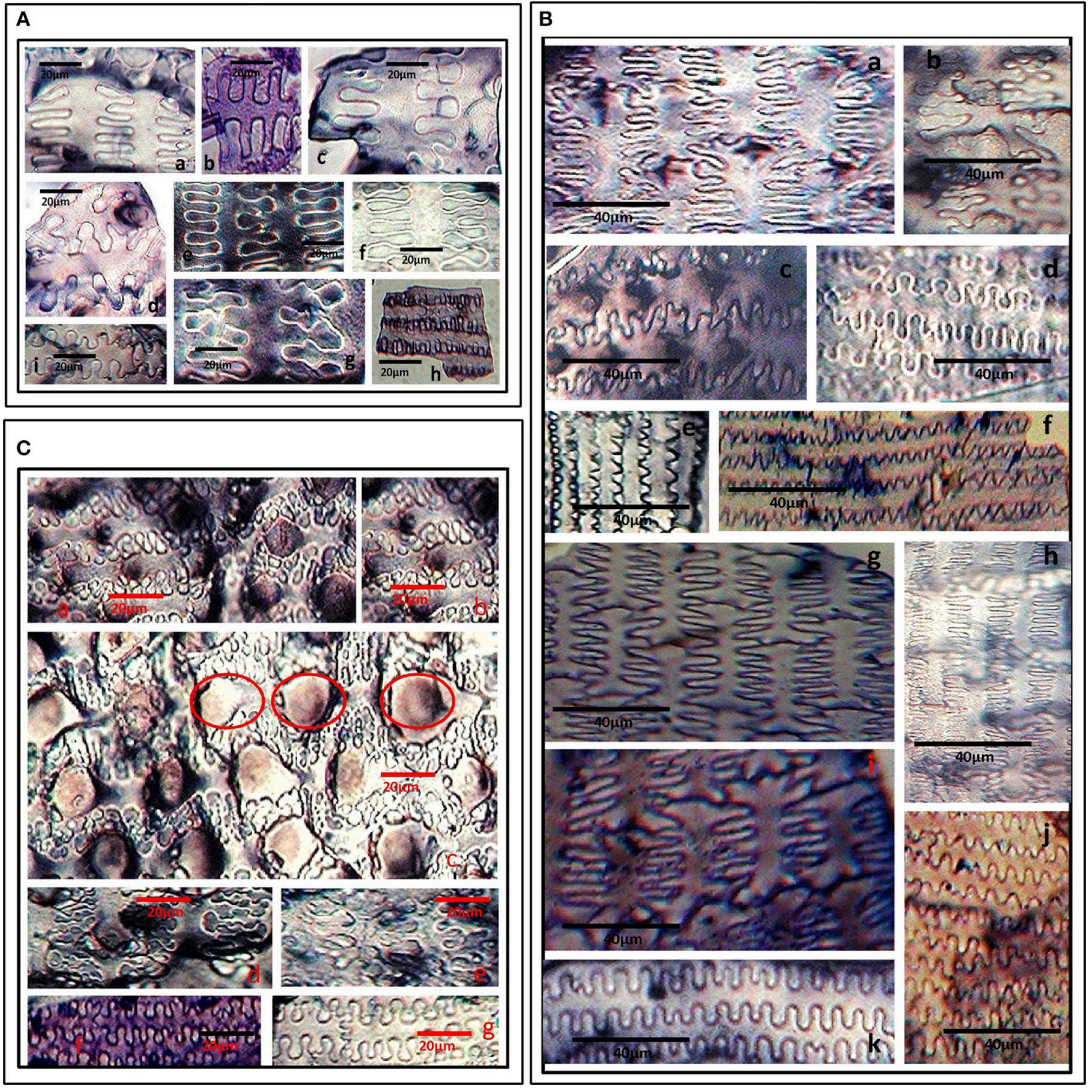
**(A)** Undulated patterns and ornamentations on the epidermal long cells of *Setaria pumila* (Poir.) Roem. & Schult. synflorescence. Columellate extensions **(a–c)**; η-I type **(d–g)**; granulate **(h**) and n-I type **(i)** type of epidermal undulation patterns. **(B)** Undulated patterns and ornamentations on the epidermal long cells of *Setaria verticillata* (L.) P. Beauv. synflorescence. η-I **(a–c)**; Ω-I **(d)**, Λ-I **(e,f)** Λ-II **(g,h)**, Λ-III **(i)** and n-I **(j)**, and n-II **(k)** type of epidermal undulation patterns. **(C)** Undulated patterns and ornamentations on the epidermal long cells of *Setaria viridis* (L.) P. Beauv. synflorescence. Ω-I **(a,b,g)**, Ω –II with papillate structures (encircled) **(c–e)** and granulate epidermal extensions **(f)**.

*S. pumila* showed columellate extensions of epidermal cells (Figures 3Aa–c) whereas they were absent in the other two species. In addition to columellate extensions, *S. pumila* showed the presence of η-I (Figures 3Ad–g), granulate (Figures 3Ah), and n-I (Figures 3Ai) type of undulated ornamentations. These types of ornamentations have been reported in some other species of *Setaria* including *S. italica*, (Lu et al., 2009; Zhang et al., 2011). In our sample, *S. verticillata* showed the presence of η-I (Figures 3Ba–c) Ω-I (Figures 3Bd), Λ-I (Figures 3Be,f) Λ –II (Figures 3Bg,h), Λ –III (Figure 3Bi) and n-I (Figure 3Bj) and n-II (Figure 3Bk). *S. viridis* showed Ω-I (Figures 3C a,b,g), Ω –II (Figures 3Cc–e) and granulate (Figures 3Cf,g). The epidermal elements also showed the presence of papillae on the surface of lemmas. Kealhofer et al. (2015) also reported the similar (Ω –II) type of epidermal undulated ornamentations in *S. viridis*.

### Phytolith morphotypes

**Figure 4:**
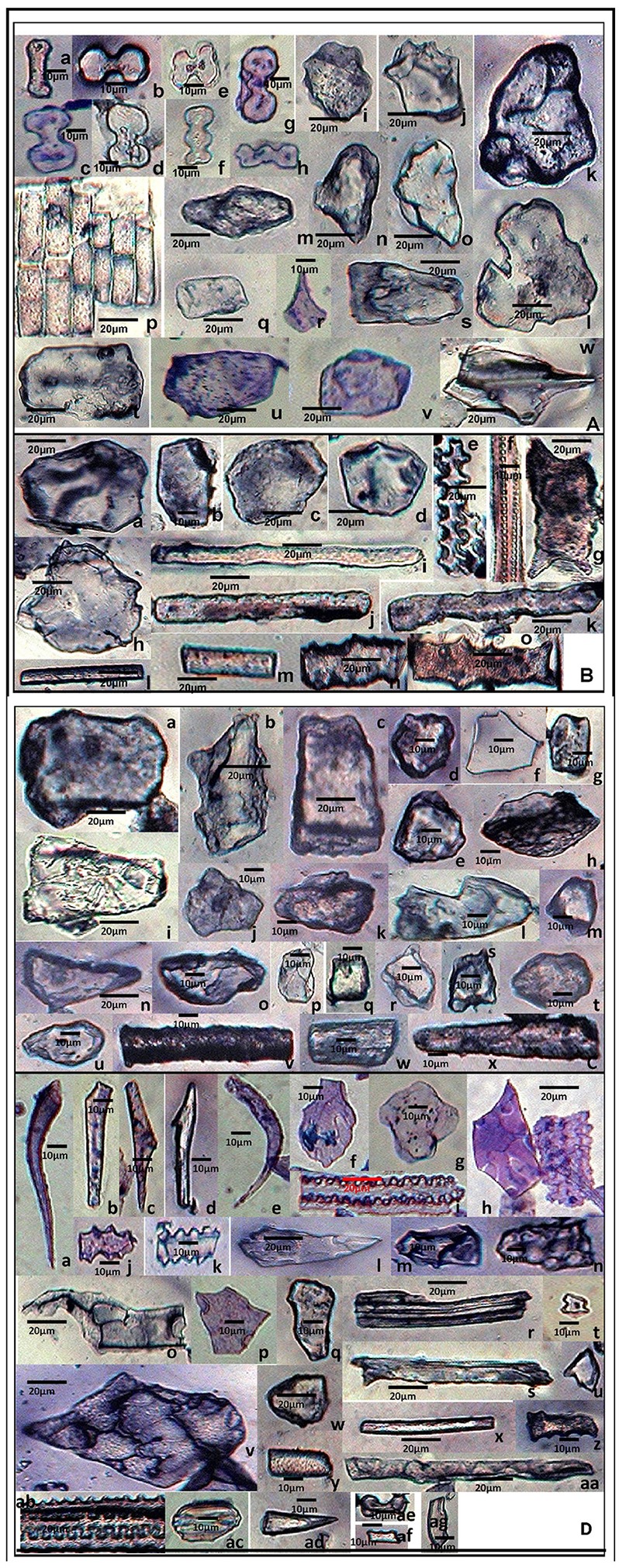
Phytolith morphotypes from various parts of *Setaria pumila* (Poir.) Roem. & Schult. **(A)** (Root): Bilobate class I **(a)**; Bilobate class VI **(b,c)**; Bilobate class V **(d,e)**; Polylobate **(f)**; Nodular bilobate **(g,h)**; Globular polyhedral **(i,j)**, Blocky irregular **(k,l)**; Oblong **(m)**; Trapezoid **(n,o)**; Rectangular **(p,q)**; Cuneiform bulliform **(r)**; Tabular irregular **(s–v)**; Scutiform **(w)**. **(B)** (Culm): Blocky polyhedral **(a,b)**; Trapezoid **(c)**; Globular polyhedral **(d)**; Echinate elongate **(e,f)**; Sinuate elongate with concave ends **(g)**; Tabular irregular **(h)**; Sinuate elongate **(i–k)**; Smooth elongate **(l,m)**; Elongate irregular **(n,o)**. **(C)** (Leaf): Tabular simple **(a)**; Blocky irregular **(b)**; Rectangular **(c)**; Globular granulate **(d,e)**; Blocky polyhedral **(f)**; Parrellepedal bulliform cells **(g)**; Trapezoid **(h–l)**; Globular polyhedral **(m)**; Clavate **(n,o)**; Cuboid **(p,q)**; Scutiform **(r,s)**; Ovate **(t,u)**; Cylindrical **(v,w)**; Smooth elongate **(x)**. **(D)** (synflorescence): Macrohairs **(a–e)**; Cuneiform bulliform **(f)**; Globular polyhedral **(g)**; Epidermal elements **(h)**; Echinate elongate **(i–k)**; Clavate **(i)**; Trapezoid **(m,n)**, Tracheid **(o)**, Blocky polyhedral **(p,q)**; Elongate irregular **(r,s)**; Horned tower **(t,u)**; Blocky irregular **(v)**; Globular granulate **(w)**; Smooth elongate **(x,y)**; Facetate elongate **(z)**; Sinuate elongate **(aa)**; Columellate elongate **(ab)**; Stomata **(ac)**; Prickle hair **(ad)**; Bilobate class I **(ae)**; Plates **(af,ag)**.

**Figure 5:**
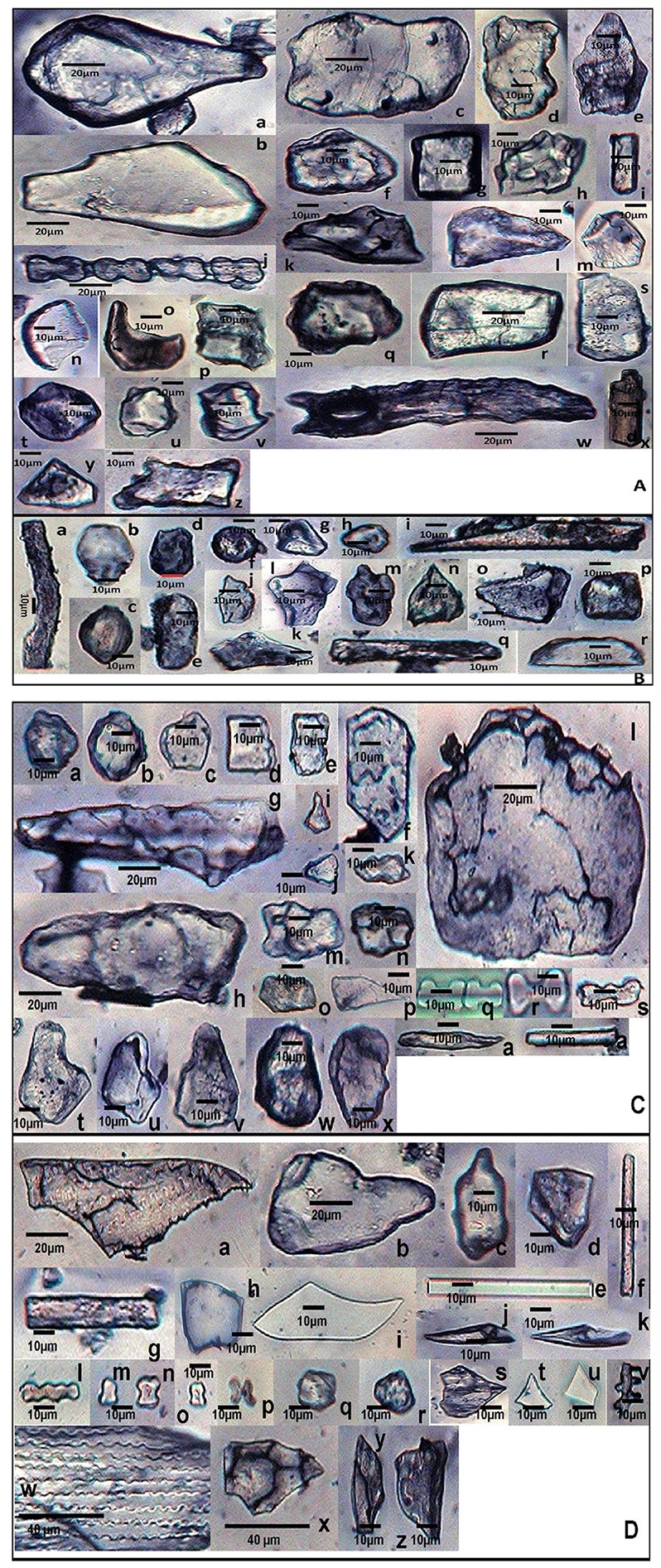
Phytolith morphotypes from various parts of *Setaria verticillata* (L.) P.Beauv. **(A)** (Root): Cuneiform bulliform **(a,b)**; Tabular simple **(c)**; Blocky irregular **(d–f)**; Cuboid **(g)**; Globular echinate **(h)**; Smooth elongate **(i)**; Bilobate class VII **(j)**; Blocky polyhedral **(k–m)**; Crescent moon **(n,o)**; Parrellepedal bulliform cells **(p,q)**; Rectangular **(r,s)**; Globular polyhedral **(t–v)**; Elongate with concave ends **(w)**; Cylindrical **(x)**; Triangular **(y)**; Trapezoid **(z)**. **(B)** (Culm): Sinuate elongate **(a)**; Ovate **(b,c)**; Blocky crenate **(d,e)**; Globular psilate **(f)**; Trapezoid **(g,h)**; Clavate **(i)**; Scutiform **(j,k)**; Blocky irregular **(l–n)**; Blocky polyhedral **(o)**; Cuboid **(p)**; Smooth elongate **(q)**; Half-moon **(r)**. **(C)** (Leaf): Globular granulate **(a,b)**; Globular polyhedral **(c)**; Rectangular **(d,e)**; Blocky polyhedral **(f)**; Elongate irregular **(g,h)**; Horned tower **(i–k)**; Tabular irregular **(l)**; Trapezoid **(m–o)**; Scutiform **(p)**; Bilobate class IV **(q)**; Bilobate class VII **(r)**; Nodular bilobate **(s)**; Cuneiform bulliform **(t–v)**; Blocky irregular **(w,x)**. **(D)** (Synflorescence): Epidermal element with columellate extensions **(a)**; Cuneiform bulliform **(b,c)**; Blocky polyhedral **(d)** Smooth elongate **(e,f)**; Rectangular **(g)**; Cuboid **(h)**; Clavate **(i)**; Acicular **(j,k)**; Polylobate irregular **(l)**; Rondel **(m–o)**; Cross **(p)**; Globular polyhedral **(q,r)**; Scutiform **(s–u)**; Columellate elongate **(v)**; Echinate elongate **(w)**; Trapezoid **(x–z)**.

**Figure 6:**
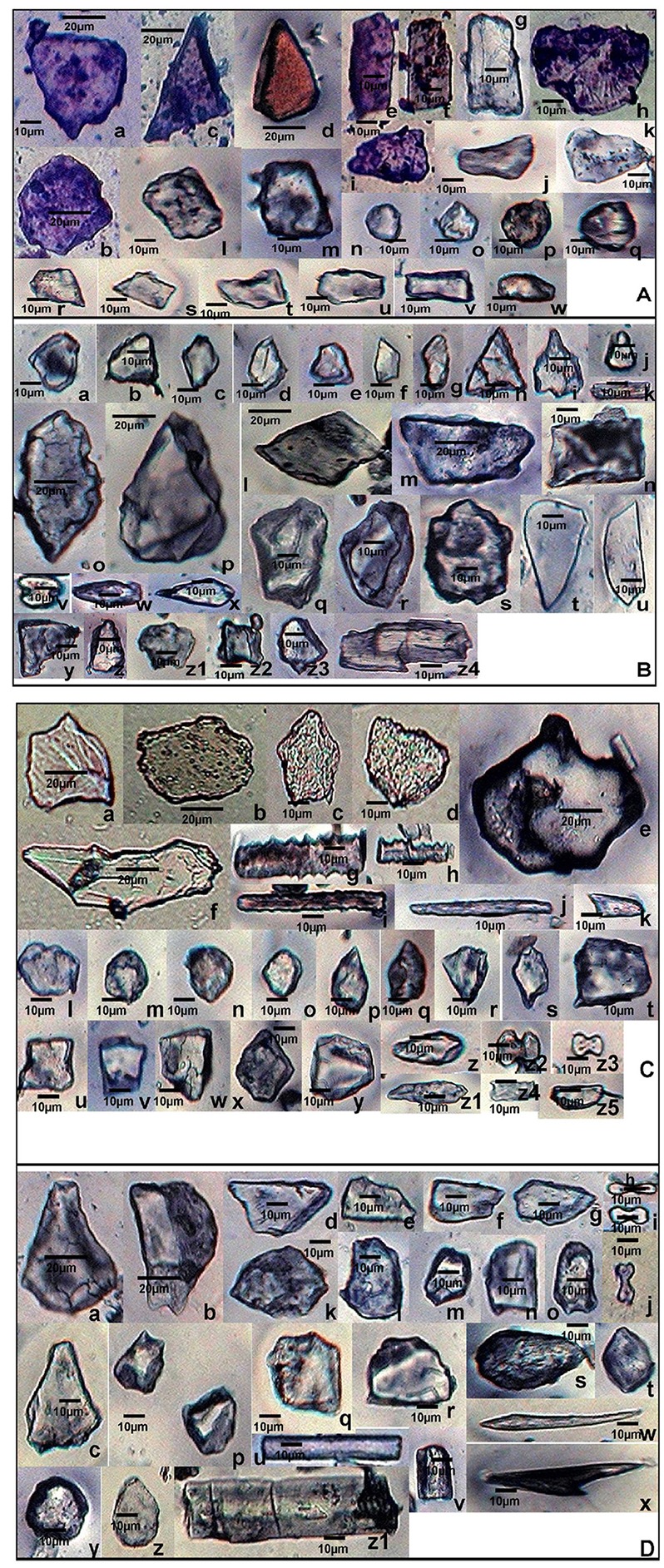
Phytolith morphotypes from various parts of *Setaria viridis* (L.) P. Beauv. **(A)** (Root): Blocky polyhedral **(a,b)**; Triangular **(c,d)**; Rectangular **(e–g)**; Blocky irregular **(h)**; Trapezoid **(i–k)**; cuboid **(l,m)**; Globular psilate **(n)**; Globular granulate **(o–q)**; Scutiform **(r,s)**; Parrellepedal bulliform cells **(t–v)**; Oblong **(w)**. **(B)** (Culm): Globular polyhedral **(a–e)**; plates **(f,g)**; Triangular **(h)**; Cuneiform bulliform **(i)**; Rondel **(j)**; Smooth elongate **(k)**; Rectangular **(l–n)**; Blocky irregular **(o,p)**; Blocky polyhedral **(q,r)**; Globular echinate **(s)**; Carinate **(t,u)**; Bilobate class VIII **(v)**; Clavate **(w,x)**; Trapezoid **(y–z1)**; Cuboid **(z2, z3)**; Elongate irregular **(z4)**. **(C)** (Leaf): Blocky irregular **(a–d)**; Tabular polyhedral **(e)**; Clavate **(f)**; Echinate elongate **(g,h)**; Sinuate elongate **(i)**; Smooth elongate **(j)**; Pickle hair **(k)**; Globular granulate **(l–o)**; Scutiform **(p–s)**; Cuboid **(t–v)**; Trapezoid **(w–y)**; Ovate **(z,z1)**; Bilobates class VII **(z2)** Bilobates class VIII **(z3)**; Plates **(z4,z5)**. **(D)** (Synflorescence): Cuneiform bulliform **(a–c)**; Blocky polyhedral **(d–g)**; Bilobate class II **(h–j)**; Blocky irregular **(k,l)**; Parrellepedal bulliform cells **(m–o)**; Globular polyhedral **(p–r)**; Ovate **(s,t)**; Smooth elongate **(u,v)**; Acicular **(w)**; Prickle hair **(x)**; Globular psilate **(y,z)**; Cylindrical **(z1)**.

**Figure 7:**
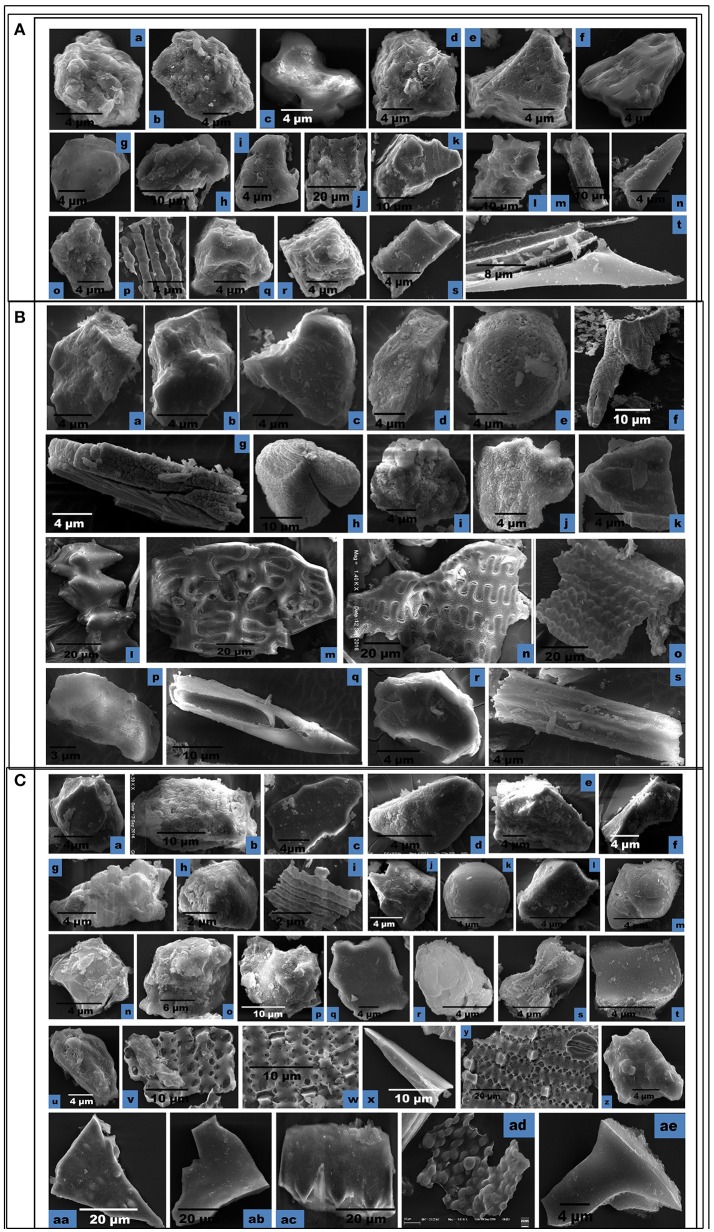
Scanning Electron Micrographs (SEM) of phytolith morphotypes from various parts of: **(A)** *Setaria pumila* (Poir.) Roem. & Schult. Root: Globular granulate **(a)** Blocky irregular **(b)**; Bilobate class V **(c)**. Culm: Globular granulate **(d)** Cuneiform bulliform **(e)**. Blocky polyhedral **(f)**; Trapezoid **(g)**; Leaf: Trapezoid **(h,i)**; Blocky irregular **(j)**; Blocky polyhedral **(k)**; Globular echinate **(l)**; Elonagate irregular **(m)**. Synflorescence: Prickle hair **(n)**; Blocky irregular **(o)**; Epidermal element with undulated ridges **(p)**; Globular polyhedral **(q,r)**; Trapezoid **(s)**; Prickle hair **(t)**. **(B)** *Setaria verticillata* (L.) P.Beauv. Root: Blocky polyhedral **(a,b)**; Cuneiform bulliform **(c)**; Trapezoid **(d)**; Globular psilate **(e)**. Culm: Scutiform **(f)**; Elongate irregular **(g)**. Leaf: Globular polyhedral **(h)**; Globular granulate **(i)**; Blocky irregular **(j)**; Blocky polyhedral **(k)**. Synflorescence: Echinate elongate **(l)**; Crenate elongate **(m)**; Columellate elongate **(n)**; Blocky papillate **(o)**; Trapezoid **(p)**; Acicular **(q)**; Blocky irregular **(r)**; Rugose elongate **(s)**. **(C)** *Setaria viridis* (L.) P. Beauv. Root: Globular polyhedral **(a)**; Blocky irregular **(b)**; Blocky polyhedral **(c,d)**; Trapezoid **(e,f)**. Culm**:** Trapezoid **(g)**. Globular polyhedral **(h)**; Epidermal element with undulated ridge (i); Blocky irregular **(j)**; Globular psilate **(k)**; Tabular polyhedral **(l)**. Leaf: Trapezoid **(m)**; Blocky polyhedral **(n)**; Globular echinate **(o,p)**; Tabular irregular **(q)**; Globular crenate **(r)**; Bilobate class V **(s)**; Tabular polyhedral **(t)**. Synflorescence: Trapezoid **(u)**; Epidermal element **(v)**; Epidermal element with silica short cells & stomata **(w,y)**; Carinate **(x)**; Globular polyhedral **(z)**; Triangular **(aa)**; Blocky polyhedral **(ab)**; Prickly elongate **(ac)**; Epidermal papillate **(ad)**; Scutiform **(ae)**.

In the present study, a cumulative total of 58 phytolith morphotypes were identified with an individual distribution of 38 in *S. pumila*, 39 in *S. verticillata*, and 41 in *S. viridis*. These morphotypes were grouped into nine broad categories namely, bulliform cells, epidermal elements, hairs, long cells, short cells, tabular types, globular types, blocky types, and tracheids (Table 1 and Figures 4–**6**, **7A–C**). The first seven categories are known to have their origin in the epidermis, blocky types in the endodermis and the last one in the vascular tissue system (Twiss et al., 1969; Lu and Liu, 2003).

Except for the blocky and globular types, phytolith morphotypes have been well reported in family Poaceae (Twiss et al., 1969; Bonnett, 1972; Prychid et al., 2004). Both blocky and globular (spherical) morphotypes are considered to be characteristic of forest trees (Runge, 1999). Even within monocots, spinulose to tabular spheres are typically associated with the arborescent (palm) family, Arecaceae (Kealhofer and Piperno, 1998) wherein these types are produced in great abundance (Albert et al., 2007). While the blocky morphotype has been reported in some grasses (Wang and Lu, 1993; Carnelli et al., 2004), we have not come across any report of the globular type in the family. However, in view of the reports of the globular type from the commelinid families, Zingiberaceae, Marantaceae, and Strelitziaceae (Tomlinson, 1956, 1961; Kealhofer and Piperno, 1998; Brilhante de Albuquerque et al., 2013) and the non-commelinid family Orchidaceae (Sandoval-Zapotitla et al., 2010), the recovery of the globular morphotype in Poaceae during the present studies was not entirely unexpected.

The present study marks a significant addition to information on phytolith profiles particularly from underground (root) parts of three species of genus *Setaria*. Most of the previous studies in grasses have documented phytoliths from above ground parts, mainly the leaf (Tomlinson, 1969; Twiss et al., 1969; Bonnett, 1972; Krishnan et al., 2000; Lu and Liu, 2003; Prychid et al., 2004; Fahmy, 2008; Barboni and Bremond, 2009; Rudall et al., 2014; Shakoor et al., 2014; Jattisha and Sabu, 2015). Only a limited number of reports are available on phytolith analysis of roots of plant species (Ezell-Chandler et al., 2006; Das et al., 2014; Soukup et al., 2014; Shakoor et al., 2016).

A comparison among the three congeneric species of *Setaria* revealed that some of the phytolith morphotypes were shared by all the three species while some others were restricted to only one or two of the three species in the present study (Table 1). At one extreme were some morphotypes which had a ubiquity value of unity, i.e. they occur in at least one plant part in all the three species. For example, bilobate class V, blocky irregular and blocky polyhedral were present at least in one plant part in all the three species and hence carried a ubiquity value of unity (Table 1). Such morphotypes have the lowest diagnostic value. Similarly, phytolith morphotypes with a ubiquity value of 0.66 indicates their presence in two out of the three species. These types could be utilized for taxonomic diagnosis and demarcation of pairs of species in the present sample from the one lacking these morphotypes (Table 1). For example, bilobate class III, columellate elongate, cross, horned tower, oblong and tabular simple demarcate *S. pumila* and *S. verticillata* from *S. viridis* in the present sample. Similar is the case with other morphotypes with ubiquity value of 0.66 between other pairs of species within the three congenerics (Table 1).

Phytolith morphotypes with ubiquity value of 0.33 indicates their presence in only one of the three studied species. Within the limited context of the present work, these phytoliths marked the individual species from the other two and helped in their taxonomic demarcation. For example, bilobate class I (Figures 4Aa,Da,e) from roots and synflorescences, polylobate (Figure 4Af) from roots, sinuate elongate with concave ends (Figure 4Bg) from culms, stomatas (Figures 4Dac) facetate elongate (Figure 4Dz), and tracheids (Figure 4Do) from synflorescences have ubiquity values of 0.33 and diagnose *S. pumila* from the other pair of species (Table 1). The “marker” phytolith morphotypes yielded by various parts of *S. verticillata* included blocky crenate (Figures 5Bd,e) from culms, crescent moon (Figures 5An,o) and elongate with concave ends (Figure 5Aw) from roots, half-moon (Figure 5Br), epidermal element with columellate extensions (Figure 5Da) and polylobate irregular (Figure 5Dl) from the synflorescences (Table 1). Similarly, the “marker” morphotypes from *S. viridis* included bilobate class II (Figures 6D-h-j), epidermal element with short silica cells and stomata, epidermal papillate, and prickly elongate (Figures 7Cw,y,ad,ac) from synflorescences, bilobate class VIII (Figure 6Bv) from culms, tabular polyhedral from the culms and leaves (Figures 6Ce, 7Cl) and carinate (Figures 6Bt,u, 7Cx) from the culms and the synflorescences (Table 1). What adds to the diagnostic significance of these morphotypes is that these were recovered from all the plant parts ranging from roots to synflorescences. Hence, the present study reiterates the necessity and significance of analysis of phytoliths from all the underground and aerial plant parts before utilizing them for taxonomic diagnosis as suggested in some earlier studies as well (Kealhofer et al., 2015; Shakoor et al., 2016). Here, it may be emphasized that these morphotypes “mark” the individual species only from the other two in the present study. An unqualified use of the term marker phytolith for the types recovered from species in the present sample would be an overstatement implying that these types diagnose these species individually from rest of the species of the foxtail grass genus *Setaria*. The full potential of phytolith types for interspecific diagnosis can only be realized after phytolith analysis of the entire genus. Similarly, “marker” types for the genus and suprageneric levels can only be identified by profiling all the taxa included in these ranks.

SEM of phytoliths of the three congenerics of *Setaria* revealed subtle differences in topography of phytolith morphotypes which was not clear in light microscopy (Figures 7A–C). SEM has helped to distinguish and segregate particular phytolith morphotypes into sub-types. For example, the globular morphotype was further resolved into globular crenate (Figure 7Cr) globular granulate (Figures 7Aa,d,Bi), globular echinate (Figures 7Al,Co,p), globular polyhedral (Figures 7Aq,r,Bh,Ca,h,z), and globular psilate (Figures 7Be,Ck) morphotypes based on the type and degree of surface ornamentations. Similarly, the tabular morphotype was segregated into tabular polyhedral (Figure 7Cl), tabular irregular (Figure 7Cq) and tabular polyhedral (Figure 7Ct). Earlier studies grouped all broad and multisided structures into trapezoid category (Piperno, 1988, 2001; Pearsall, 2000). But recent studies have distinguished two more categories within the trapezoid morphotype *viz.*, blocky polyhedral and blocky irregular morphotypes (Traoré et al., 2014). We have also recognized blocky irregular (Figures 7Ab,o,Bj,r,Cb) and blocky polyhedral (Figures 7Af,k,Ba,b,Cc,d,n,ab) morphotypes. Additionally, SEM has revealed the interlocking patterns between epidermal elements (Figures 7Cv,w,y). It has also revealed the presence of silica short cells embedded with the epidermal elements (Figure 7Cw).Thus, SEM has been employed as an effective tool in elucidation of ultrastructural features of phytolith morphotypes and their classification into subtypes that have further helped in demarcation of the grass species under reference.

The coefficient of association of phytolith morphotypes based on Pearson's association revealed highest association among overground parts (Supplementary Table 4). The strongest association was found among the leaf and synflorescence of *S. pumila* and *S. viridis* whereas *S. verticillata* showed significantly lower values of association (Supplementary Table 4). The highest values of coefficient of association between leaf and synflorescence could be attributed to the anatomical similarities of leaf and synflorescence bracts that produce phytoliths. Similarly, insignificant association between the underground and overground parts could be explained by the anatomical, histological and physiological differences among these plant parts and hence the phytolith morphotypes produced by them.

**Figure 8:**
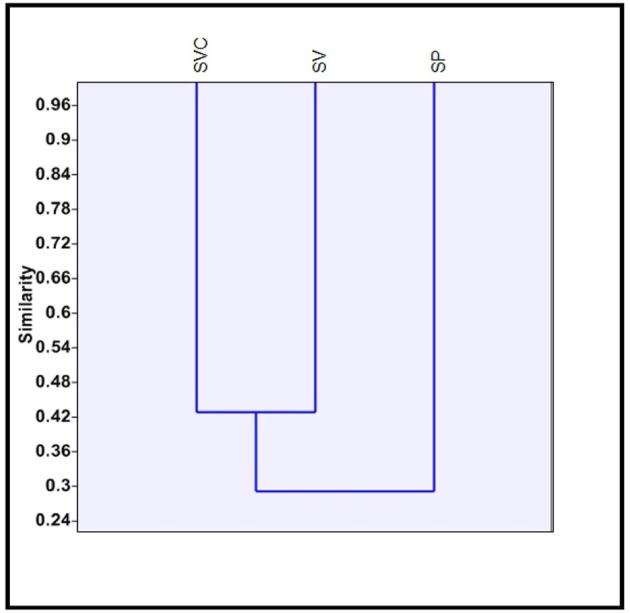
Clustering of three species of *Setaria* P. Beauv. based on presence/absence data of bilobate phytolith morphotypes. [SP, *Setaria pumila* (Poir.) Roem. & Schult.; SVC, *Setaria verticillata* (L.) P. Beauv.; SV, *Setaria viridis* (L.) P. Beauv.].

Clustering of species on the presence/ absence data of bilobate classes, using Jaccard's similarity index was carried out. *S. pumila* belongs to one clade of *Setaria* whereas the other two species belong to the other clade (Doust and Kellogg, 2002). A similar trend was observed in clusters from the totality of morphotypes (Figure 8). *S. pumila* stood apart from the other two species as it has 33% similarity of phytolith profile with *S. verticillata* and 28.57% with *S. viridis*. However, *S. verticillata* and *S. viridis* showed 42.85% similarity and were grouped together (Figure 8).

### Frequency distributions and morphometric measurements

Several studies in the past have utilized data on morphotypes for taxonomic characterization and identification of plant species (Twiss et al., 1969; Lau et al., 1978; Hodson and Sangster, 1988; Ollendorf et al., 1988; Whang et al., 1998; Krishnan et al., 2000; Ponzi and Pizzolongo, 2003; Piperno, 2006). However, recent studies have enlarged the scope of phytolith research by including data on morphometric measurements and frequency distributions of phytolith morphotypes for taxonomic demarcation of species down the taxonomic hierarchy from family, genus, and species levels (Strömberg, 2009; Jattisha and Sabu, 2012; Tripathi et al., 2013; Szabo et al., 2014; Shakoor et al., 2016; Ball et al., 2017; Out and Madella, 2017).

**Figure 9:**
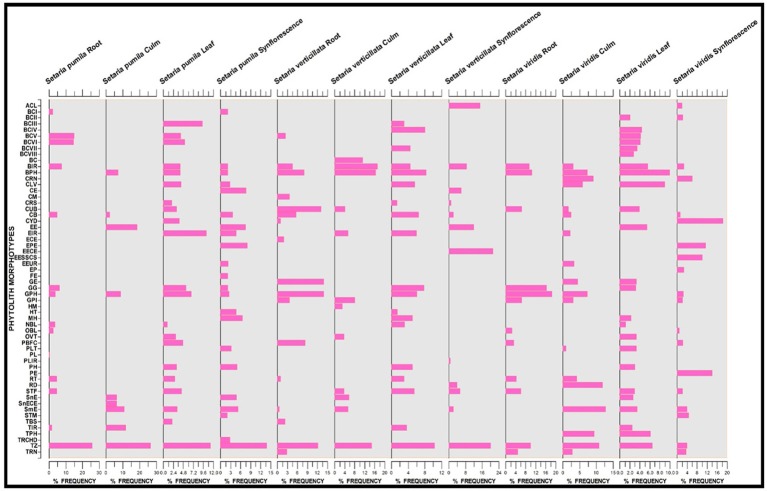
Stratigraphic diagram showing percentage frequency of different phytolith morphotypes. (Description of phytolith morphotypes from Table 1).

*Setaria* spp. showed considerable differences in the frequency distribution of various phytolith morphotypes (Figure 9). The most frequent in all the three species were the trapezoids. However, they differ significantly within and between the species with 19.47% frequency in *S. pumila*, 14.38% in *S. verticillata* and 7.91% in *S. viridis* (*p* ≤ 0.05; χ^2^-test). Acicular morphotypes present in both *S. verticillata* and *S. viridis* differed many fold in terms of their percentage frequency with 15.17% in the former and 2.18% in the later species. Bilobate classes also differ significantly with respect to frequency distributions. For example, bilobate class III were present in the leaves of *S. pumila* and *S. verticillata* with highly variable percentage frequency values of 9.44% and 3.10% respectively (*p* ≤ 0.05; χ^2^-test). Similarly, bilobate class IV occurred in the leaves of *S. verticillata* and *S. viridis* with a percentage frequency of 8.10 and 4.39% respectively (*p* ≤ 0.05; χ^2^-test). Similarly, other phytolith morphotypes revealed significant differences in percentage frequency providing a definite clue that frequency of occurrence of phytolith morphotypes provides an additional evidence for taxonomic characterization apart from qualitative differences in phytolith types (Figure 9).

Apart from frequency distributions, morphometric data on size dimensions and shape descriptors of morphotypes also revealed significant differences between the species (Supplementary Tables 5A–C). In the present analysis, we included data on size parameters (length, width, area and perimeter) and one shape descriptor, the aspect ratio. Further, length and width of the shank of bilobate types have been employed as additional characteristics to classify the bilobates into various subtypes in order to further supplement taxonomic diagnosis of species (Supplementary Table 3). The use of multivariate statistical approaches like principal component analysis has been recommended and employed in earlier studies for taxonomic demarcation of species (Benvenuto et al., 2015; Pearsall, 2015; Ball et al., 2016).

Joint PCA analysis of morphometric parameters of phytoliths from different parts of the three species led to overcrowding of the data and did not help in diagnosis of species. However, PCA analysis of morphometric parameters of phytoliths from different parts individually proved useful in taxonomic demarcation of the species. PCA analysis of root phytoliths clearly separated the three species on the basis of surface areas of different morphotypes (Supplementary Figures 1a,b). *S. pumila* was demarcated on the basis of surface areas of blocky irregular and tabular irregular, *S. verticillata* by blocky polyhedral and *S. viridis* by area of trapezoid morphotypes as revealed by PCA results of component 1 and 2 (Supplementary Figure 1a). However, the PCA plot between component 1 and 3 revealed more clear demarcation than obtained from components 1 and 2 (Supplementary Figures 1b). PCA analysis of phytolith morphotypes of culm of the three species brought about the taxonomic demarcation of species on the basis of the area of smooth elongates for *S. verticillata*, and tabular irregular for *S. pumila* (Supplementary Figure 2). Similarly, PCA analysis of leaf and synflorescence phytolith morphotypes of the three species lent further support to taxonomic analysis of the three species of *Setaria* (Supplementary Figures 3, 4).

### Transmission electron microscopy

**Figure 10:**
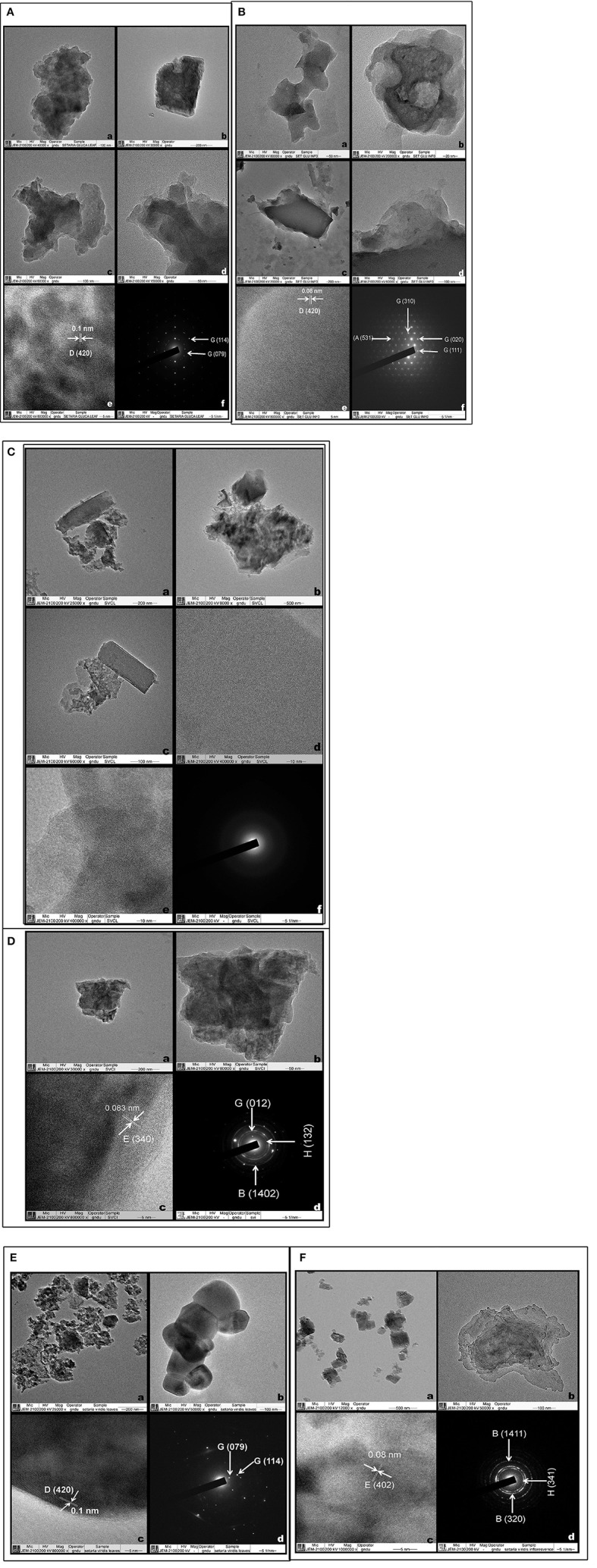
Transmission electron microscopy of Phytoliths **(A,B)**: *Setaria pumila* (Poir.) Roem. & Schult. Leaf **(A) (a–d)** Clusters and agglomerates of silica **(e)** HRTEM **(f)** SAED patterns (Figures in parenthesis indicate hkl values and for description of alphabets refer Supplementary Table 8) and Synflorescence **(B) (a–d)** (Clusters and agglomerates of silica **(e)** HRTEM **(f)** SAED patterns (Figures in parenthesis indicate hkl values and for description of alphabets refer Supplementary Table 8). **(C,D)** *Setaria verticillata* (L.) P. Beauv. Leaf **(C) (a–c)** Clusters and agglomerates of silica **(d–e)** HRTEM **(f)** SAED patterns and Synflorescence **(D) (a–b)** Clusters and agglomerates of silica **(c)** HRTEM **(d)** SAED patterns (Figures in parenthesis indicate hkl values and for description of alphabets refer Supplementary Table 8). **(E,F)** *Setaria viridis* (L.) P. Beauv. Leaf **(E) (a,b)** Spherical silica particles **(c)** HRTEM **(d)** SAED patterns (Figures in parenthesis indicate hkl values and for description of alphabets refer Supplementary Table 8) and Synflorescence **(F) (a,b)** Cubic and agglomerated silica **(c)** HRTEM **(d)** SAED patterns (Figures in parenthesis indicate hkl values and for description of alphabets refer Supplementary Table 8).

TEM allows visualization and microstructural examination through a combination of high magnification and resolution. It helped to distinguish various physical states including amorphous from the crystalline and helped to study their atomic planes, (columns of atoms in crystals). TEM images of phytolith morphotypes from leaves and synflorescences of *S. pumila* and *S. verticillata* showed macroscopic clusters and agglomerates of silica that were not distinguished into particles at nanoscale regime (Figures 10Aa–d,Ba–d,Ca–c,Da,b). However, phytoliths from leaves and synflorescences of *S. viridis* revealed silica particles of spherical and cubic morphologies of nanoscale regime and were clustered (Figures 10Ea,b,Fa,b). The presence of spherical and cubic nanoparticle clusters in the latter species clearly demarcates it from the other two congenerics. Gonzalez-Espindola et al. (2014) reported clusters and agglomerates of phytoliths as well as spherical particles of nanoscale regime from the leaves of the grass species *Stenotaphrum secundatum*. Palanivelu et al. (2014) reported agglomerated particles of silica nanoparticles from rice hulls collected from different geographical locations.

High resolution transmission electron microscopy (HRTEM) revealed the presence of ordered interplanar atomic layers of Si–O, Si–O–Si bonds in all the species except in the leaf phytoliths of *S. verticillata* (Figures 10Ae,Be,Dc,Ec,Fc), which did not possess regular ordering of local clusters of Si–O and the silica bodies were completely amorphous (Figures 10Bd,e). HRTEM analysis of phytoliths from leaves of *S. pumila* and *S. viridis* revealed microcrystalline structures with an interplanar distance (d-spacing) of 0.1 nm which was indicative of the presence of tetragonal cristobalite polymorph of silica (Figures 10Ae,Fc). Similarly, silica from the synflorescences of all the three species revealed microcrystalline structures with a difference of interplanar distance which was 0.08 nm for *S. pumila* and *S. viridis* and 0.083 nm for *S. verticillata*. These distances correspond to tetragonal stishovite polymorphs (Figures 10Be,Dc,Fc) whose formation was favored by the presence of localized crystallization centers such as extraneous cations dispersed throughout the siliceous phytoliths (Mann and Perry, 1986). The link got substantiated and explained by the presence of cations like Al^2+^, Ba^2+^, Fe^2+^,Ca^2+^,Cu^2+^, Mg^2+^, Na^+^, and K^+^ as revealed by SEM-EDX analysis of phytoliths (Supplementary Table 6).

Selected area electron diffraction (SAED) reveals the chemical composition of different mineral phases by their different patterns generated by the impact of X-rays and fast moving electrons. Phytoliths from the leaves of *S. pumila* and *S. viridis* revealed well defined single crystalline lattices that could be resolved to hexagonal and orthorhombic lattices of SiO_2_. (Figures 10Af,Ed) that were continuous and unbroken in the former but lacked grain boundaries in the latter (cf. Reid et al., 2011). The amorphous structure of phytoliths was revealed by an absence of SAED patterns (Figure 10C). Similarly, phytoliths from synflorescences of *S. pumila* revealed single crystal lattices corresponding to SiO_2_ (with cubic, tetragonal and orthorhombic morphologies) and zeolites with a cubic lattice system (Figure 10B). The SAED pattern of synflorescences of *S. verticillata* and *S. viridis* showed well defined rings confirming their polycrystalline nature (Figures 10Dd,Fd). The SAED patterns of phytoliths from *S. verticillata* correspond to orthorhombic ferrierite and tridymite and anorthic SiO_2_ polymorphs. Similarly, SAED patterns of silica from *S. viridis* correspond to orthorhombic ferrierite and tridymite (Figure 10Fd). Apart from taxonomic resolution, the formation of nanoscale silica particles during dry ashing of the plant material has applications in nanotechnology, particularly synthesis of metal silicates (Neethirajan et al., 2009; Qadri et al., 2015).

### Biosilica content

Grasses deposit silica in varied amounts in different plant parts ranging from 1 to 11% (Jones and Handreck, 1967). In the present study, the three species of *Setaria* revealed considerable differences in terms of ash and silica content in their parts (Supplementary Figure 5). The species showed higher values of ash and silica in their foliar parts with 21.06% ash and 11.62% silica in *S. pumila*, 19.87% ash and 9.23% silica in *S. verticillata* and 16.43% ash and 6.24% silica in *S. viridis*. The ash and silica content in other parts were in the order of, synflorescences>roots>culms. Higher amounts of silica in the leaves and synflorescences of grasses are well reported (Lanning and Eleuterius, 1981, 1987, 1989). The differential amounts of silica within and between different parts of the plant body have been correlated to the differences in the targeted cellular sites of silicification. For example, in roots endodermal cells have been proved to be the usual targets of silicification while in the aerial parts of the plant body different epidermal cells and associated structures as well as the cells of vascular bundles, and the spaces between the cortical cells are believed to be the targeted sites of silicification (Kumar et al., 2017; Kumar and Elbaum, 2018).

Our results indicated significantly higher silica content in the leaves of the presently studied *Setaria* species as compared to some other species of the genus. For example a much lower amount (6.06%) was reported in *S. magna* Griseb. (Hodson et al. (1982)) and other members of tribe Paniceae (1.04% for *Panicum reptans* L., 3.7% for *Digitaria macroblephara* (Hack.) Paoli) and related tribes (1.34% for *Imperata cylindrical* (L.) Raeusch. and 2.7% for *Themeda triandra* Forssk.) of the subfamily Panicoideae (Lanning and Eleuterius, 1989; Quigley and Anderson, 2014).

The variation in silicification rates in underground and aerial parts (particularly leaf and synflorescence bracts) are known to be controlled by a multitude of extrinsic (availability of silica and water in the soil) and intrinsic factors including the extent and nature of silicon transporters and channels, sink strength and the functional anatomy of various plant parts (Motomura et al., 2002; Ma and Yamaji, 2006; Honaine and Osterrieth, 2011). Besides these factors of control, higher levels of silicification in leaf laminae and the synflorescence bracts of aerial plant parts have been correlated with higher evapotranspiration rates in these parts. Once absorbed, silica is transported *via* xylem to various plant parts through the transpiration stream. As water evaporates during transpiration, silicic acid solutes are progressively concentrated resulting in super-saturated concentrations of Si(OH)_4_ and deposition in tissues as amorphous silica in the form of phytoliths; the extent of supersaturation being controlled by the concentration of silicic acid in soil water (Jones and Handreck, 1965; Rosen and Weiner, 1994; Raven, 2003; Exley, 2015).

### Elemental composition

Though mainly siliceous in nature, phytoliths deposit many other elements in addition to silicon and oxygen in varying proportions during the course of their development (Shakoor et al., 2016). The elemental composition of phytolith morphotypes is reported to be controlled by species characteristics, geochemistry and prevailing environmental conditions (Bujan, 2013; Kamenik et al., 2013; Hodson, 2016). However, the elemental composition of phytoliths in association with their morphology has proved useful for taxonomic diagnosis of species. Elemental composition has been shown to be stable enough to serve as definitive evidence of palaeo-environments by providing clues to the type of the soil in which a given species grew (Kamenik et al., 2013; Hodson, 2016).

The presence of different elements in phytolith morphotypes of the present samples reflect the availability of elements in the soil (Supplementary Table 7). However, the present study revealed some species-specific elements as well. The elemental composition of rhizospheric soil samples from the three sampling sites (Figure 1) revealed a cumulative number of fourteen (14) elements (aluminum (Al), carbon (C), calcium (Ca), copper (Cu), iron (Fe), magnesium (Mg), sodium (Na), phosphorous (P), potassium (K), oxygen (O), silicon (Si), sulfur (S), titanium (Ti), and zinc (Zn). Species wise characterization of the soil revealed 11 elements (Al, Ca, C, Fe, Mg, O, K, Si, Na, Ti, and Zn excluding Cu, P, and S from the cumulative list) from sampling sites of *S. pumila*, 10 elements (excluding Cu, P, S, and Zn) from the soil supporting *S. verticillata* whereas the rhizospheric soil samples from the *S. viridis* sampling site revealed 12 elements (excluding Na and Zn from the cumulative list).

**Figure 11:**
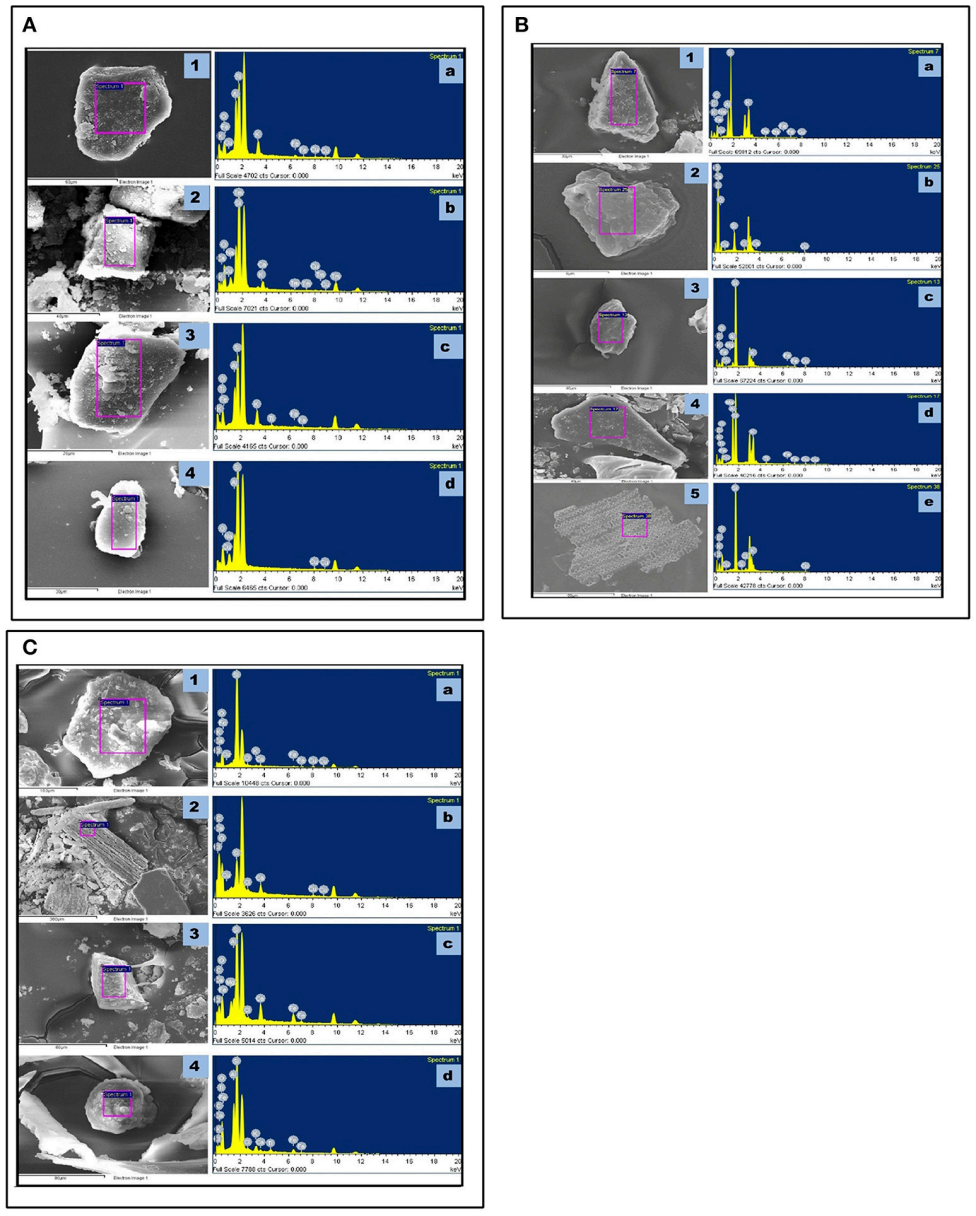
SEM-EDX spectra of phytoliths isolated from different parts: **(A)** *Setaria viridis* (L.) P. Beauv. Root **(1-a)**; Culm **(2-b)**; Leaf **(3-c)**; and Synflorescence **(4-d)**. **(B)** *Setaria verticillata* (L.) P.Beauv. Root **(1-a)**; Culm **(2-b)**; Leaf **(3-c,4-d)**, and Synflorescence **(5-e)**. **(C)** *Setaria pumila* (Poir. Roem. & Schult. Root **(1-a)**; Culm **(2-b)**; Leaf **(3-c)**; and Synflorescence **(4-d)**.

**Figure 12:**
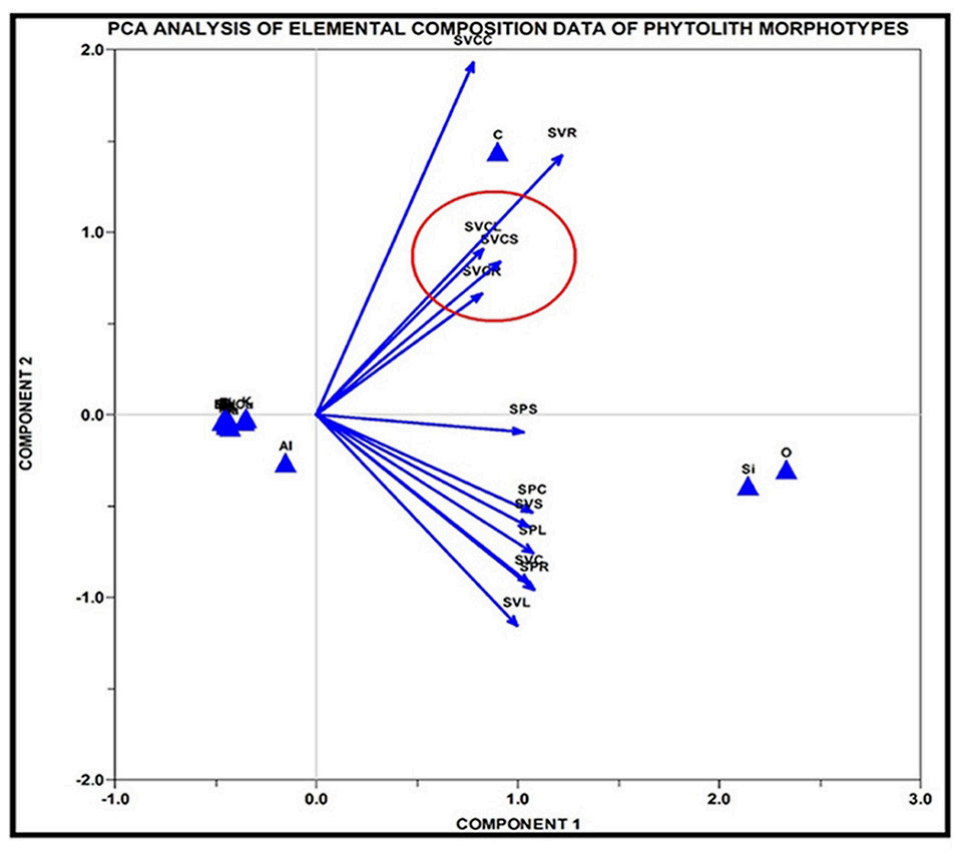
PC analysis of elemental composition of data of phytolith morphotypes of *Setaria* spp. SPR, Setaria pumila root; SPC, *Setaria pumila* culm; SPL, *Setaria pumila* leaf; SPS, *Setaria pumila* synflorescence; SVCR, *Setaria verticillata* root; SVCC, *Setaria verticillata* culm; SVCL, *Setaria verticillata* leaf; SVCS, *Setaria verticillata* synflorescence; SVR, *Setaria viridis* root; SVC, *Setaria viridis* culm; SVL, *Setaria viridis* leaf; SVS, *Setaria viridis* synflorescence.

SEM-EDX analysis of the phytolith morphotypes from different parts of the three species revealed a cumulative total of 16 elements with 12 in *S. pumila* 14 in *S. verticillata* and 11 in *S. viridis* (Figures 11A–C and Supplementary Table 6). A comparison of elemental composition data of soil samples and phytolith morphotypes revealed that soil geochemistry controls the composition of phytoliths. However, some elements were present in phytolith samples in traces but were not detected in soil samples. For example chlorine (Cl) was detected in phytoliths from all parts of *S. pumila* and *S. verticillata*. Similarly barium (Ba), copper (Cu), and sulfur (S) were detected in the latter named species and rubidium (Rb) and sodium (Na) in *S. viridis*. This unexpected difference in elemental composition of soil samples and phytoliths could be attributed to some sort of “accumulation” of these elements in the living cells producing phytoliths. Most importantly, some elements were unique to one or the other species: barium (Ba), phosphorous (S), and sulfur (S) were detected in *S. verticillata* and rubidium (Rb) in *S. viridis* Principal Component Analysis (PCA) of elemental composition data from different parts of the three congeneric species led to demarcation of *S.‘verticillata* from the other two congenerics with the first two components explaining 97.12% (85.12% component 1 + 15% component 2) variation in the data set (Figure 12). The present study has revealed higher atomic and weight percentage values for carbon (C), oxygen (O), and silicon (Si) in phytoliths whereas other elements were present in considerably lesser amounts. The occlusion of carbon in phytoliths has been compared to its sequestration in cellulose and lignin (Parr and Sullivan, 2005). However, EDX analysis revealed the element form of carbon in phytoliths rather than the organic form.

Biomineralization of silica in plants is known to ameliorate metal (Al, Cu, Fe) and salinity stress (Okuda and Takahashi, 1962; Matoh et al., 1986; Cocker et al., 1998; Yeo et al., 1999). The deposition of metals like Al, Cu, Fe in phytoliths possibly alleviates the toxicity associated with these elements. Similarly, salinity stress seemed to be ameliorated by the bioaccumulation of silicophytoliths as revealed by K, Ca, and Mg in phytoliths (Anala and Nambisan, 2015). The segregation and compartmentalization of phytoliths embodying Si and other minerals has made isolation of these elements possible (Raven, 1983). Thus, deposition and immobilization of these toxic elements in the silicification process may be a strategy of plant species to get rid of these materials via their transport along the transpirational route and final occlusion in phytoliths.

### X-ray diffraction analysis

Silica exists in diverse polymorphs and sub-morphs; crystalline forms include alpha and beta-quartz, cristobalite, tridymite, coesite, keatite, and stishovite. Amorphous silica has the same composition as SiO_2_ but has a random structure of the crystal lattice. The presence of both types in our specimens can be attributed to the transformation of amorphous silica into different crystalline polymorphs during dry ashing of the material (Holm et al., 1967).

**Figure 13:**
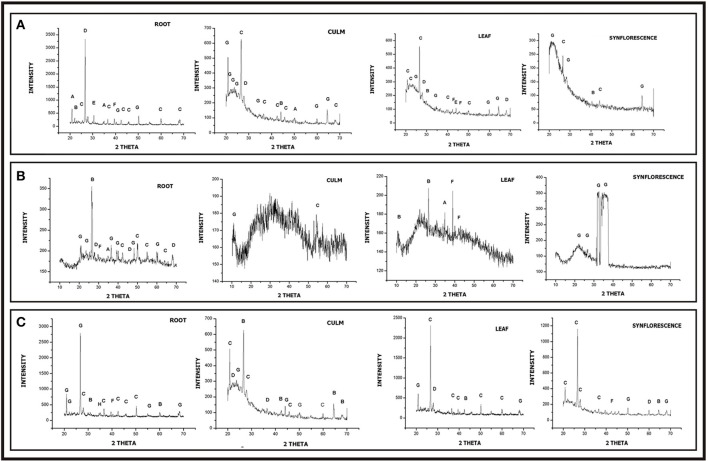
XRD diffraction spectra of phytoliths isolated from different parts of *Setaria* spp. **(A)** *Setraia pumila* **(B)** *Setaria verticillata* **(C)** *Setaria viridis* (for description of peak points, refer to Supplementary Table 8).

Powder diffractograms of phytoliths isolated from underground and aerial parts of *Setaria* showed peaks characteristic of different crystalline polymorphic phases (Figures 13A–C). The most frequent phases were silicon dioxide (SiO_2_) from all the parts of the species (except the leaf of *S*. verticillata) and quartz (except in leaves and synflorescences of *S. verticillata*. The other phases present in all the three species (at least in one of the parts) included zeolites, tridymite, stishovite, ferririte, coesite and cristobalite (Figures 13A–C and Supplementary Table 8). However, stishovite was diagnostic of roots and leaves of *S. pumila* whereas ferririte was restricted only to the roots of *S. viridis*, suggesting a role in taxonomic diagnosis as already reported for some of the grass species (Gonzalez-Espindola et al., 2010, 2014; Shakoor et al., 2016).

The polymorphic phases have been known to have an identical chemical composition (SiO_2_) but different physical properties and lattice symmetries. They show distinct lattice systems ranging from anorthic (triclinic), through monoclinic, orthorhombic, hexagonal, cubic, and tetragonal. The present studies lend further credence to the existence of polymorphic silica in plants (Ollendorf et al., 1988; Piperno, 1988, 2006; Lu and Liu, 2003; Lu et al., 2009; Zhang et al., 2011; Szabo et al., 2015; Ge et al., 2016). The diffractogram of phytoliths of *S. viridis* (root) and *S. pumila* (root and culm) showed a unique peak corresponding to ferrierite and zeolite respectively. (Figures 13A,C). Ferrierite is a zeolite (aluminosilicate mineral) that binds a number of cations viz., Na^+^, K^+^, Ca^2+^, Mg^2+^ etc. The presence of these phases can be explained by elemental composition data.

**Figure 14:**
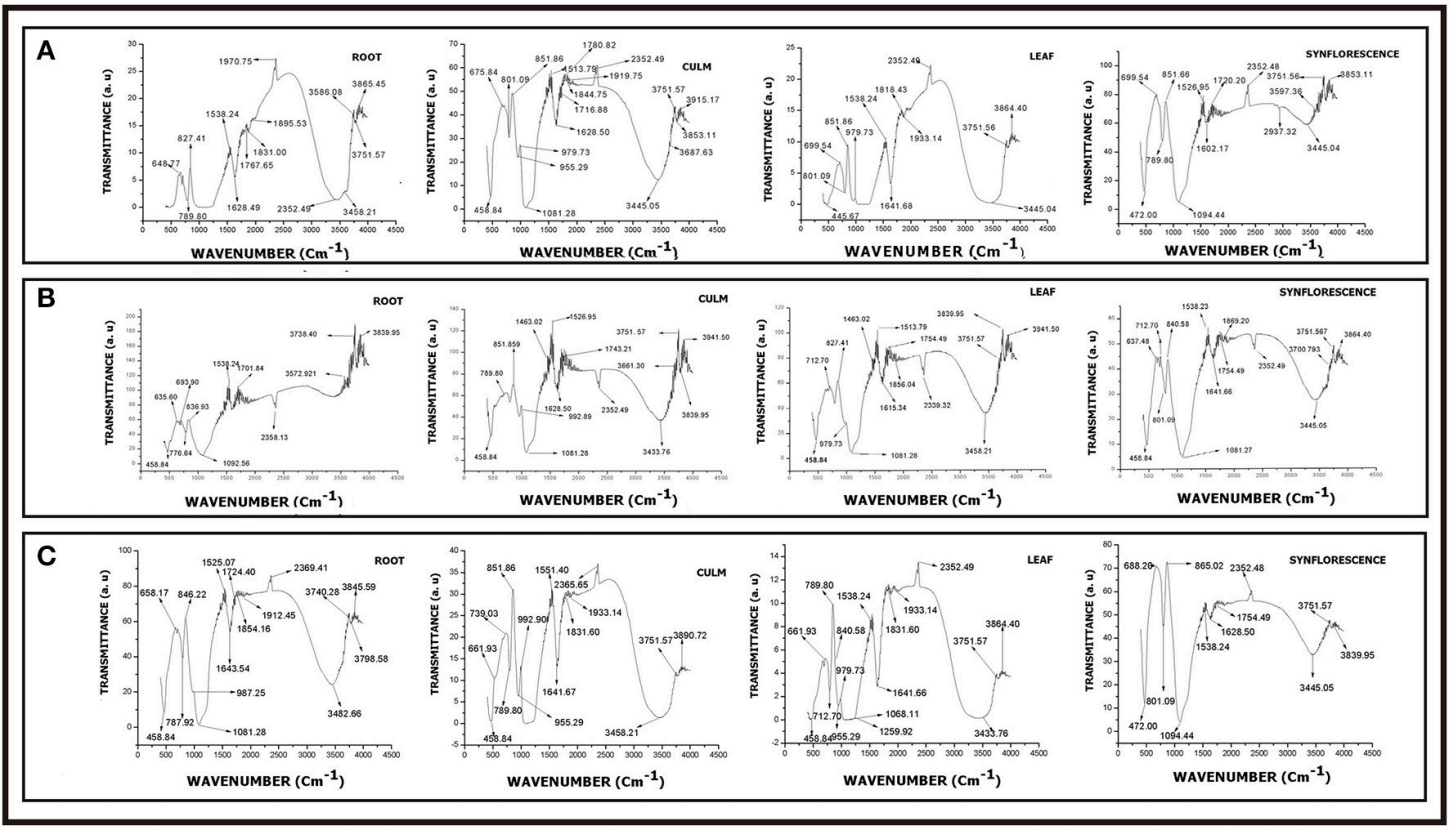
FTIR spectra of phytoliths from different parts of *Setaria sps*. **(A)** *Setaria pumila* **(B)** *Setaria verticillata* **(C)** *Setaria viridis* (for description of peak points, refer to Supplementary Table 9).

Further, the FTIR analysis revealed a peak of Aluminosilicate minerals in these species, thus supporting our XRD results (Figures 14A,C). Earlier, Kow et al. (2014) confirmed the shift from amorphous to crystalline phases of silica in cogon grass (*Imperata cylindrica* (L.) P. Beauv.) in the presence of potassium (K). Similarly, the presence of other minerals like, Na, Ca, Mg, K etc. in phytoliths from the different parts of these congeneric species could afford a possible explanation (acting as a controlling factor) for the presence of different crystalline polymorphic phases of silica. Such an association is further indicated by the presence of only amorphous silica in the phytoliths from the culms of *S. verticillata* that harbor a smaller number of elements (only 4 besides C & H) as compared to phytoliths from other parts of the plant body (Figure 13B and Supplementary Table 7).

### FT-IR spectroscopy

FTIR spectroscopy of silica from different parts of *Setaria* spp. revealed several peaks that could be assigned to different structural units of silica with varied vibrational degrees of freedom (Figures 14A–C and Supplementary Table 9). The peaks between 445.67–472.00 cm^−1^, 637.48–699.54 cm^−1^, 712.70–801.08 cm^−1^, 1080.06–1094.44 cm^−1^, 1602.17-1616.24 cm^−1^, 1628.50–1641.66 cm^−1^, 2339.32–2366.49 cm^−1^, and 3346.27–3597.36 cm^−1^ present in all the three species (Figures 14A–C and Supplementary Table 9) have earlier been variously ascribed to deformation vibration of O–Si–O group (Bertoluzza et al., 1982), symmetrical vibration of Si–O–Si (Gopal et al., 2004), symmetric vibration of Si–O (Brinker et al., 1990), asymmetric vibration of Si–O–Si (Karunakaran et al., 2013; Mourhly et al., 2015), inplane stretching vibration of C-C (Ou and Seddon, 1997), deformation vibration of H–O–H (Socrates, 2001), inplane stretching vibration of Si–C (Socrates, 2001) and O–H/Si–OH bonds (Brinker et al., 1990) bonds. Peaks between 530.39–563.18, 1164.92, 1218.93, 1323.08–1332.72, 1743.21–1933.14, 2825.52, and 3006.82–3271.05 cm^−1^ characteristic of *S. verticillata* (L.) P. Beauv. (Figure 14B and Supplementary Table 9) could be ascribed to stretching vibration of O–Si (SiO_2_ defect) (Brinker et al., 1990), asymmetric vibration of Si–O–Si (Duran et al., 1986), inplane stretching of free Si–O (Chmel et al., 2005), symmetric deformation vibration of Si–R (Socrates, 2001), deformation vibration of R (alkyl group), symmetric vibration of C–H (Gunzler and Gremlich, 2002), and stretching vibration of O–H bonds (Brinker et al., 1990). Similarly, peaks at 1463.02 and 1701.84 cm^−1^ characteristic of *S. viridis* (Figures 14C and Table 10) could be ascribed to asymmetric and symmetric deformation vibrations of hydrocarbons (–CH_3_-CH_2_)– (Watling et al., 2011) and inplane stretching vibrations of Si–C bonds.

## Conclusions

Within the context, scope and parameters of reference samples used in the present work, the three congenerics of *Setaria* revealed a degree of similarity in phytolith profiles but each was found to be well demarcated from the other in the group by “unique” morphotypes and their characteristic assemblages and structures. The bilobate morphotypes aptly illustrate phytolith-assisted taxonomic demarcation of the three species. In the present study, eight variants of the bilobate morphotype were recognized on the basis of the length of the shanks (the interconnecting segment between the lobes) and the shape of the outer margin of the lobes. *S. pumila* showed three of the eight structural variants of the bilobate phytoliths (III, V, and VI) in the costal region on the adaxial surfaces. In the same location, *S. viridis* also showed three of these variants (II, IV, and V) whereas *S. verticillata* had only two of them (VII and IV). Thus bilobate classes were found to be highly conserved and useful for identification of grass species. Quadrihedral and hexahedral cystoliths (calcium oxalate crystals) on the adaxial epidermal surfaces of *S. verticillata* emerged as another diagnostic feature of the species (a first report for the foxtail grass genus *Setaria*)*S. verticillata* was also marked out by the presence of a new undulation type, (the Λ-lambda with three variants *viz*. Λ-I, Λ-II, and Λ-III) in the long epidermal cells.

Besides qualitative differences, the present samples of the three species also revealed interspecific variations in frequency distribution and morphometric measurements of various morphotypes. For example, the frequency of trapezoids was significantly different in these species: 19.47% in *S. pumila*, 14.38% in *S. verticillata*, and 7.91% in *S. viridis* (*p* ≤ 0.05; χ^2^-test). Acicular morphotypes were present in both *S. verticillata* and *S. viridis* but differed many fold in their percentage frequency (15.17 and 2.18% respectively).

Principal Component Analysis of morphometric parameters of phytoliths from different parts of the plant body proved useful in taxonomic demarcation of the species. PCA of root phytoliths clearly separated the three species on the basis of the surface area of different morphotypes. *S. pumila* was demarcated on the basis of the surface area of blocky irregular and tabular irregular, *S. verticillata* by the surface area of blocky polyhedral and *S. viridis* by the area of trapezoid morphotype.

TEM revealed three valuable distinguishing parameters of phytoliths namely, micro-structural details, degree of amorpho-crystalline nature and inter-atomic planer distances in crystalline samples. Secondly, ultramicroscopy has proved useful in comparing and collating phytolith profiles from different parts of the plant body to develop phytolith signatures for each species. SAED patterns revealed by TEM showed the polycrystalline nature of silica in the synflorescences of *S. verticillata* and *S. viridis* whereas single crystal systems were reported in other parts of the three species. Thirdly, indexing of SAED patterns revealed silica polymorphism. The polymorphs of silica revealed by TEM were further confirmed by XRD patterns, particularly the ferrierite in *S. viridis* (root) and zeolite in *S. pumila* (root and culm).

The elemental composition of phytolith morphotypes from different parts of the present samples of the three species has revealed a cumulative total of 16 elements with 12 in *S. pumila* 14 in *S. verticillata* and 11 in *S. viridis*. A comparison of elemental composition of soil samples and phytolith morphotypes revealed that soil geochemistry controls the composition of phytoliths. Powder diffractograms of phytoliths revealed a number of polymorphic phases of silica. Stishovite was diagnostic of roots and leaves of *S. pumila* whereas ferririte was restricted only to the roots of *S. viridis*, thus strengthening a case for their role in taxonomic diagnosis as already reported for some other grass species.

FTIR analysis has revealed diversity of functional groups and their modes of vibrations with some groups being exclusively species specific. *S. verticillata* showed stretching vibration of O–Si (SiO_2_ defect), asymmetric vibration of Si–O–Si, inplane stretching of free Si–O bond, symmetric deformation vibration of Si–R, deformation vibration of R (alkyl group), symmetric vibration of C–H and stretching vibration of O–H bonds. Similarly, groups characteristic of *S. viridis* include asymmetric and symmetric deformation vibrations of hydrocarbons (–CH_3_-CH_2_) – and inplane stretching vibrations of Si–C bonds.

The multiproxy approach employed in the present work has led to anatomical and physico-chemical characterization of the phytoliths produced by the present samples of three related species of the foxtail genus *Setaria* Phytolith analysis seems to confirm the comparatively isolated position of *S. pumila* in the present triumvirate of species. *S. pumila* was marked by two unique bilobate types compared to only one each in the other two species, the absence of polycrystalline silica in the synflorescences and the presence of the polymorphic silica as stishovite in the roots and the leaves. Clustering of species using Jaccard's similarity index for presence/absence data of the entire data set of phytolith morphotypes also revealed that *S. pumila* had a low similarity (33%) of phytolith profiles with *S. verticillata* and *S. viridis* (28.57%). However, *S. verticillata* and *S. viridis* showed much higher similarity (42.85%) and were grouped together (Figures 8). A plausible explanation may lie in the difference in the centers of origin of *S. pumila* (Africa) and the other two species (Asia).

Even though the full potential of phytoliths in understanding the taxonomy and phylogeny of the foxtail grass genus (*Setaria*) must come through future research involving an assessment of inter-population and intra-population variations and construction of representative master profiles for each species, the paper has made an initial contribution. We have made plant collections from single locations and homogenized the material part-wise but this limitation has been partly made good by following a multiproxy and multi-organ approach in carrying out the present work. In the larger context of plant systematics, concerted and coordinated efforts of a multidisciplinary nature are required to develop integrated and robust phytolith profiles of different groups of plants and their application in the characterization and diagnosis of plant taxa.

## Author contributions

MB collected the material, conducted leaf epidermal studies, and wrote the initial draft of the manuscript. SS and PB carried out experimental work. AS designed the work, guided the conduct of experiments and checked the final manuscript.

### Conflict of interest statement

The authors declare that the research was conducted in the absence of any commercial or financial relationships that could be construed as a potential conflict of interest.
