# Supplementary material for: Taxonomic Demarcation of Setaria pumila (Poir.) Roem. & Schult., S. verticillata (L.) P. Beauv., and S. viridis (L.) P. Beauv. (Cenchrinae, Paniceae, Panicoideae, Poaceae) From Phytolith Signatures
Source: Front Plant Sci. 2018 Jun 22;9:864. doi: 10.3389/fpls.2018.00864 (PMC6024613; doi:10.3389/fpls.2018.00864)
Supplement: Supplementary file 1 [file Table_1.pdf]

Supplementary Table 1: Morphological and morphometrical characters of species of *Setaria* P. Beauv.

S.No.	Characteristic	*S. pumila*	*S. verticillata*	*S. viridis*
1.	Habit	Caespitose annual	Loosely tufted annual	Tufted annual
2.	Culms	Geniculately ascending	Decumbent	Erect or geniculate
3.	Culm length (cm)	20-130	10-100	10-60
4.	Length of leaf (cm)	7-30	5-22	6.5-25
5.	Width of leaf (mm)	2-10	4-18	4-12
6.	Leaf sheaths	Glabrous or sparsely hairy	Glabrous or pubescent	Glabrous or papillose/pilose
7.	Ligule	Fringe of hairs	Ciliate	Fringe of hairs
8.	Ligule length (mm)	1-2	0.5-1	1.2-2
9.	Leaf sheath margins	Glabrous	Scabrous	Densely ciliate
10.	Length of inflorescence (cm)	9.5	12	13
11.	Spikelets	Ovate	Elliptic	Ellipsoid
12.	Spikelet length (mm)	1.5-3.5	1.5-2-5	2-2.5
13.	Lower glume	Orbicular	Obtuse	Oblate
14.	Length of lower glume (mm)	0.3-0.7	0.6-1.2	0.5-1
15.	Lower glume apex	Acute	Acute	Obtuse
16.	Upper glume	Ovate	Boat shaped	Elliptic
17.	Length of upper glume (mm)	0.4-0.7	1.8-2.4	2.2.5
18.	Upper glume apex	Acute	Acute	Obtuse
19.	Lower glumes nerves	3-nerved	3-5 nerved	3-5 nerved
20.	Upper glume	Ovate	Elliptic	
21.	Upper glume nerves	5-nerved	7 nerved	5-nerved
22.	Lemma	Ovate	Ovate	Oblong
23.	Lemma apex	Obtuse	Acute	Obtuse
24.	Lemma surface	Strongly rugose	Finely rugose	Finally rugose
25.	Length of lemma (mm)	1.5-3.5	1.5-2.3	2-3
26.	Palea	Ovate	Elliptic	Ovate
27.	Length of anther (mm)	0.7-0.8	0.7-1.1	0.3-0.6
28.	Shape of caryopsis	Ovoid to ovate	Ovate to obovate	Elliptic
29.	Caryopsis (mm)	1.3-1.5	1	1.5-2.3

S Supplementary Table 2: Presence/absence matrix for diagnostic phytolith morphotypes in leaf epidermis of *Setaria* spp.

S.No.	Species	Surface View	Region	Phytolith rows	Phytolith morphotypes												Cystoliths	
					BLI	BLII	BLIII	BLIV	BLV	BLVI	BLVII	BLVIII	NBL	CR	PH	MH	QDH	HXD
1.	***Setaria pumila*** **(Poir.) Roem. & Schult.**	Adaxial	Costal	1-3	------	------	+	------	+	+	------	------	+	-----	+	------	------	-----
			Intercostal	-----	------	------	------	------	------	------	------	------	------	-----	+	------	------	------
		Abaxial	Costal	1-3	------	------	+	------	+	------	------	------	------	+	+	------	------	------
			Intercostal	-----	------	------	------	------	------	------	------	------	------	------	+	------	------	------
2.	***Setaria verticillata*** **(L.) P. Beauv.**	Adaxial	Costal	3-6	------	------	------	+	------	------	+	------	+	+	+	------	+	+
			Intercostal	-----	------	------	------	------	------	------	------	------	------	------	------	+	+	+
		Abaxial	Costal	1	------	------	+	+	------	------	------	------	+	------	------	+	------	------
			Intercostal	-----	------	------	------	------	------	------	------	------	------	------	------	+	------	------
3.	***Setaria viridis*** **(L.) P. Beauv.**	Adaxial	Costal	1-4	------	+	------	+	+	------	------	------	+	------	+	------	------	------
			Intercostal	1	------	------	------	------	------	------	------	------	------	------	------	------	------	------
		Abaxial	Costal	1	------	------	------	+	------	+	------	------	+	------	------	+	------	------
			Intercostal	------	------	------	------	------	------	------	------	------	------	------	------	------	------	------

Supplementary Table 3: Morphological classification of bilobate phytolith morphotypes.

Bilobate phytolith description	Class No.	Size dimensions	
		**Length of Shank**	**Width of Shank**
Bilobates shaped like phone receiver.	I	>8 µm	<4 µm
Bilobates with long shank and concave lobes.	II	>5 µm	<5 µm
Bilobates with long shank and convex lobes.	III		
Bilobates with long shank and flat lobes.	IV		
Bilobates with short shank and concave lobes	V	<5 µm	≥4 µm
Bilobates with short shank and Convex lobes.	VI		
Bilobates with short shank and flat lobes	VII		
Stipa type bilobates.	VIII	<3 µm	≥5 µm

Supplementary Table 4 : Coefficient of association among the phytolith morphotypes from different parts of the three congeneric species of *Setaria* P. Beauv. based on the Pearson's coefficient.

Species		*Setaria pumila*			
		**Root**	**Culm**	**Leaf**	**Synflorescence**
Setaria pumila	**Root**	1	0.0077	0.0379	0.0541
	**Culm**	0.0077	1	0.0603	0.0503
	**Leaf**	0.0379	0.0603	1	0.1046
	**Synflorescence**	0.0541	0.0503	0.1046	1

Species		*Setaria verticillata*			
		**Root**	**Culm**	**Leaf**	**Synflorescence**
Setaria verticillata	**Root**	1	0.0074	0.0557	0.0557
	**Culm**	0.0074	1	0.0088	0.0012
	**Leaf**	0.0557	0.0088	1	0.00036
	**Synflorescence**	0.0557	0.0012	0.00036	1

Species		*Setaria viridis*			
		**Root**	**Culm**	**Leaf**	**Synflorescence**
Setaria viridis	**Root**	1	0.0579	0.00637	0.0579
	**Culm**	0.0579	1	0.0161	0.000093
	**Leaf**	0.00637	0.0161	1	0.2513
	**Synflorescence**	0.0579	0.000093	0.2513	1

Supplementary Table 5A: Morphometric measurements of phytolith morphotypes of *Setaria pumila* (Poir.) Roem. & Schult.

S. No	Phytolith Morphotypes	*Setaria pumila* (Poir.) Roem. & Schult.																			
		ROOT					CULM					LEAF					SYNFLORESCENCE				
		Length	Width	Area	Prem	A/R	Length	Width	Area	Prem	A/R	Length	Width	Area	Prem	A/R	Length	Width	Area	Prem	A/R
1.	Bilobate class I	28.37±0.56	8.46±0.44	236.71±7.6	79.54±1.64	3.71±0.11	------------	------------	------------	------------	------------	------------	------------	------------	------------	------------	32.68±2.05	10.60±0.51	300.77±22.70	92.07±4.70	3.66±0.33
2.	Bilobate class III	------------	------------	------------	------------	------------	------------	------------	------------	------------	------------	33.80±1.07	14.17±0.56	438.70±23.76	103.00±2.98	2.43±0.06	------------	------------	------------	------------	------------
3.	Bilobate class V	12.76±0.40	5.18±0.29	105.81±8.34	55.61±2.02	3.16±0.21	------------	------------	------------	------------	------------	14.98±0.82	9.61±0.51	155.64±4.15	67.13±2.12	1.45±0.53	------------	------------	------------	------------	------------
4.	Bilobate class VI	16.87±0.37	3.29±0.54	72.98±7.57	45.90±2.02	3.35±0.23	------------	------------	------------	------------	------------	14.42±0.51	10.88±0.40	151.10±1.72	56.45±14	1.34±0.05	------------	------------	------------	------------	------------
5.	Blocky irregular	87.67±9.89	54.60±4.5	4102.4±776.86	252.76±25.61	1.53±0.12	------------	------------	------------	------------	------------	81.73±10.23	41.48±2.40	3096±487.83	236.58±25.27	1.86±0.18	61.66±7.95	37.18±5.50	2468.83±587.84	203.14±27.57	1.58±0.18
6.	Blocky polyhedral	------------	------------	------------	------------	------------	80.70±5.80	48.63±2.88	3365.73±394.48	227.71±13.16	1.60±0.08	62.88±3.84	43.55±4.41	2440.97±309.27	193.61±11.84	1.36±0.06	60.13±6.46	34.78±2.09	2213.64±347.31	200.82±21.57	1.53±0.11
7.	Clavate	------------	------------	------------	------------	------------	------------	------------	------------	------------	------------	58.88±7.00	16.58±2.15	892.19±183.60	142.90±14.60	3.58±0.30	62.40±4.34	11.19±1.30	584.18±55.56	144.58±8.90	5.20±0.70
8.	Columellate elongate	------------	------------	------------	------------	------------	------------	------------	------------	------------	------------	------------	------------	------------	------------	------------	70.74±8.76	51.05±3.54	2534.63±485.31	445.43±41.30	1.54±0.14
9.	Cross	------------	------------	------------	------------	------------	------------	------------	------------	------------	------------	9.47±0.62	5.43±0.80	48.90±3.25	34.40±2.20	1.39±0.03	------------	------------	------------	------------	------------
10.	Cuboid	------------	------------	------------	------------	------------	------------	------------	------------	------------	------------	48.44±7.90	28.14±2.46	1667.85±342.43	152.74±17.15	1.47±0.07	------------	------------	------------	------------	------------
11.	Cuneiform bulliform	57.20±5.06	30.21±1.64	1405.80±226.48	157.48±13.39	1.75±0.09	55.62±6.61	34.23±2.12	1810.34±219.43	174.74±13.26	1.44±0.14	------------	------------	------------	------------		40.27±1.94	23.72±1.80	840.70±105.54	118.08±7.40	1.46±0.16
12.	Cylindrical	------------	------------	------------	------------	------------	------------	------------	------------	------------	------------	109.47±19.93	20.45±1.80	2187.74±447.93	251.80±39.94	5.37±1.08	------------	------------	------------	------------	------------
13.	Echinate elongate	------------	------------	------------	------------	------------	103.42±12.38	16.83±2.35	1635.91±233.67	248.00±23.34	7.14±1.45	------------	------------	------------	------------	------------	71.77±12.50	10.43±1.46	844.98±198.78	208.14±36.78	6.40±0.87
14.	Elongate irregular	------------	------------	------------	------------	------------	------------	------------	------------	------------	------------	150.79±20.31	22.05±1.12	3396.15±403.47	344.51±39.86	6.37±1.40	98.83±7.29	12.96±1.57	1151.14±158.21	226.73±15.74	8.05±1.05
15.	Epidermal element	------------	------------	------------	------------	------------	------------	------------	------------	------------	------------	------------	------------	------------	------------	------------	73.64±5.29	37.95±2.33	2621.87±307.98	217.14±14.33	1.66±0.10
16.	Epidermal element with undulated ridges	------------	------------	------------	------------	------------	------------	------------	------------	------------	------------	------------	------------	------------	------------	------------	66.76±8.41	40.16±4.34	2380.33±468.60	341.55±21.14	1.93±0.24
17.	Facetate elongate	------------	------------	------------	------------	------------	------------	------------	------------	------------	------------	------------	------------	------------	------------	------------	128.56±12.16	23.172±3.50	3188.67±653.57	302.78±28.60	5.62±1.07
18.	Globular granulate	40.46±2.76	34.65±2.34	1183.64±181.38	130±8.2	1.17±0.03	------------	------------	------------	------------		32.15±1.77	27.38±1.66	791.28±83.19	110.73±6.00	1.26±0.04	36.67±3.86	29.17±2.90	1315.07±237.03	141.53±13.63	1.18±0.05
19.	Globular polyhedral	37.27±3.42	29.84±3.36	994.32±201.31	117.76±11.93	1.33±0.05	42.53±3.21	34.41±2.02	1252.62±168.17	131.64±8.86	1.24±0.02	38.09±2.44	30.66±1.56	967.72±76.88	122.25±5.09	1.30±0.04	32.47±1.50	28.52±2.22	1015.99±127.95	120.19±7.20	1.18±0.05
20.	Horned tower	------------	------------	------------	------------	------------	------------	------------	------------	------------	------------	------------	------------	------------	------------	------------	14.53±1.46	8.44±0.60	130.73±14.55	49.78±3.26	1.45±0.12
21.	Macrohairs	------------	------------	------------	------------	------------	------------	------------	------------	------------	------------	------------	------------	------------	------------	------------	68.47±3.42	9.40±0.45	558.80±37.80	153.70±8.31	5.98±0.58
22.	Nodular bilobate	28.47±2.23	11.21±0.86	335.68±35.64	86.12±5.20	2.62±0.25	------------	------------	------------	------------	------------	24.37±2.35	9.15±1.75	276.35±14.50	71.35±3.40	2.42±0.70	------------	------------	------------	------------	------------
23.	Oblong	48.55±3.36	20.76±1.64	858.68±122.83	124.44±9.07	2.31±0.11	------------	------------	------------	------------	------------	------------	------------	------------	------------	------------	------------	------------	------------	------------	------------
24.	Ovate	------------	------------	------------	------------	------------	------------	------------	------------	------------	------------	49.18±3.05	21.53±0.99	893.71±101.91	126.40±8.45	2.18±0.08	------------	------------	------------	------------	------------
25.	Parallelepipedal bulliform cell	------------	------------	------------	------------	------------	------------	------------	------------	------------	------------	42.20±3.23	24.91±2.19	1088.84±133.12	136.46±10.22	1.71±0.04	------------	------------	------------	------------	------------
26.	Plates	------------	------------	------------	------------	------------	------------	------------	------------	------------	------------	------------	------------	------------	------------	------------	25.21±1.05	8.19±0.77	229.41±18.28	66.57±2.72	2.72±0.21
27.	Polylobate	28.91±0.60	9.68±0.57	264.63±6.20	90.17±1.97	2.94±0.06	------------	------------	------------	------------	------------	------------	------------	------------	------------	------------	------------	------------	------------	------------	------------
28.	Prickle hair	------------	------------	------------	------------	------------	------------	------------	------------	------------	------------	54.65±5.05	6.15±1.15	337.43±32.45	117.70±6.75	5.23±0.47	50.66±4.38	7.35±0.55	379.29±49.06	114.85±9.96	4.86±0.36
29.	Rectangular	34.47±3.14	11.58±2.10	445.30±151.39	90.79±9.84	3.26±0.32	------------	------------	------------	------------	------------	64.87±9.48	25.63±2.75	1861.39±525.67	181.27±23.19	2.77±0.50	------------	------------	------------	------------	------------
30.	Scutiform	53.13±4.11	22.35±1.96	1074.33±105.22	145.12±7.63	2.14±0.23	------------	------------	------------	------------	------------	44.60±3.81	18.55±1.12	817.67±124.64	124.23±11.17	2.24±0.18	------------	------------	------------	------------	------------
31.	Sinuate elongate	------------	------------	------------	------------	------------	106.21±8.51	15.45±1.82	1533±126.70	245.29±16.08	7.80±1.13	------------	------------	------------	------------	------------	82.90±9.60	12.40±1.58	1097.13±213.60	199.60±20.07	6.36±0.72
32.	Sinuate elongate with convex ends	------------	------------	------------	------------	------------	93.34±6.43	23.80±1.63	2100.78±141.00	248.94±9.53	3.98±0.50	------------	------------	------------	------------	------------	------------	------------	------------	------------	------------
33.	Smooth elongate	------------	------------	------------	------------	------------	83.38±7.26	11.88±1.50	1000±167.74	192.54±15.78	8.14±1.20	75.57±8.38	16.63±1.90	1317.62±192.90	183.90±14.30	4.60±0.61	67.84±8.88	9.60±0.86	714.40±152.22	156.33±18.66	6.50±0.62
34.	Stomata	------------	------------	------------	------------	------------	------------	------------	------------	------------	------------	------------	------------	------------	------------	------------	43.25±2.33	22.88±1.15	1075.05±108.98	128.74±7.81	1.82±0.62
35.	Tabular simple	------------	------------	------------	------------	------------	------------	------------	------------	------------	------------	74.43±10.15	45.45±5.01	3697.09±7.55	232.85±24.36	1.42±0.11	------------	------------	------------	------------	------------
36.	Tabular irregular	94.78±9.24	59.62±3.06	4975.20±691.44	281.76±21.97	1.54±0.12	86.29±8.04	61.13±4.81	4546.06±803.50	257.02±21.16	1.32±0.05	------------	------------	------------	------------	------------	------------	------------	------------	------------	------------
37.	Tracheid	------------	------------	------------	------------	------------	------------	------------	------------	------------		------------	------------	------------	------------	------------	69.33±5.48	19.29±1.44	1369.38±171.46	185.12±12.08	3.50±0.31
38.	Trapezoid	48.01±4.5	32.0±3.22	1348.29±207.97	141.81±10.26	1.44±0.06	57.98±4.04	37.62±2.98	1965.53±311.80	172.96±11.86	1.48±0.09	54.34±7.47	31.81±3.03	1587.24±359.95	157.85±19.90	1.52±0.10	44.31±6.28	25.60±2.11	1249.20±201.38	147.32±13.53	1.74±13.53

The entries in the Table indicate Mean±SE & [-------]=Absence of phytolith morphotype; A/R=Aspect ratio & Prem=Perimeter

Supplementary Table 5B: Morphometric measurements of phytolith morphotypes of *Setaria verticillata* (L.) P. Beauv.

S.No	Phytolith Morphotypes	*Setaria verticillata* (L.) P. Beauv.																			
		ROOT					CULM					LEAF					SYNFLORESCENCE				
		Length	Width	Area	Prem	A/R	Length	Width	Area	Prem	A/R	Length	Width	Area	Prem	A/R	Length	Width	Area	Prem	A/R
1.	Acicular	-------------	-------------	-------------	-------------	-------------	-------------	-------------	-------------	-------------	-------------	-------------	-------------	-------------	-------------	-------------	60.38±4.92	11.77±0.99	573.70±183.90	137.50±10.61	0.85±0.02
2.	Bilobate class III	-------------	-------------	-------------	-------------	-------------	-------------	-------------	-------------	-------------	-------------	25.56±0.80	10.98±0.37	245.46±7.15	76.12±2.10	2.20±0.23	-------------	-------------	-------------	-------------	-------------
3.	Bilobate class IV	-------------	-------------	-------------	-------------	-------------	-------------	-------------	-------------	-------------	-------------	26.90±0.69	8.40±0.48	182.83±19.00	69.80±3.71	3.22±0.12	-------------	-------------	-------------	-------------	-------------
4.	Bilobate class V	19.04±1.23	15.01±0.90	260.48±19.73	70.23±2.98	1.65±0.04	-------------	-------------	-------------	-------------	-------------	-------------	-------------	-------------	-------------	-------------	-------------	-------------	-------------	-------------	-------------
5.	Bilobate class VII	-------------	-------------	-------------	-------------	-------------	-------------	-------------	-------------	-------------	-------------	18.70±0.25	11.50±0.46	237.93±8.18	70.67±1.38	1.75±0.06	-------------	-------------	-------------	-------------	-------------
6.	Blocky crenate	-------------	-------------	-------------	-------------	-------------	66.43±2.03	39.00±1.25	2216.27±119.36	119.87±4.24	1.57±0.04	-------------	-------------	-------------	-------------	-------------	-------------	-------------	-------------	-------------	-------------
7.	Blocky irregular	52.28±4.80	33.07±3.05	1438.85±218.50	152.14±12.21	1.57±0.12	57.54±3.36	32.54±1.84	1752.86±169.97	168.24±8.90	1.60±0.10	60.94±3.78	34.78±2.41	1734.47±171.83	174.182±8.87	1.68±0.10	90.09±13.84	43.04±4.00	4134.25±972.16	274.80±36.54	1.82±0.17
8.	Blocky polyhedral	67.94±6.40	39.14±2.36	2290.68±318.17	196.06±15.93	1.54±0.09	70.61±8.67	42.52±7.0	2806.85±943.66	204.28±27.0	1.45±0.05	67.23±6.33	37.80±3.19	2393.21±384.14	201.39±16.09	1.63±0.08	-------------	-------------	-------------	-------------	-------------
9.	Clavate	-------------	-------------	-------------	-------------	-------------	-------------	-------------	-------------	-------------	-------------	76.31±8.38	16.04±2.00	1057.57±223.56	179.76±20.62	4.85±0.65	-------------	-------------	-------------	-------------	-------------
10.	Columellate elongate	-------------	-------------	-------------	-------------	-------------	-------------	-------------	-------------	-------------	-------------	-------------	-------------	-------------	-------------	-------------	100.79±9.44	26.45±12.45	1279.55±119.30	391.00±35.5	6.68±0.95
11.	Crescent moon	55.72±4.50	32.22±2.47	1626.51±261.91	162.14±12.46	1.53±0.06	-------------	-------------	-------------	-------------	-------------	-------------	-------------	-------------	-------------	-------------	-------------	-------------	-------------	-------------	-------------
12.	Cross	-------------	-------------	-------------	-------------	-------------	-------------	-------------	-------------	-------------	-------------	8.40±0.50	6.80±0.30	53.40±3.56	43.40±3.33	1.02±0.02	10.63±0.40	6.37±0.37	68.62±4.56	39.29±2.17	1.26±0.04
13.	Cuboid	25.44±3.15	19.55±1.90	541.87±95.81	91.54±7.59	1.29±0.08	42.01±5.94	33.01±3.51	1379.57±319.73	137.93±17.07	1.34±0.08	-------------	-------------	-------------	-------------	-------------	-------------	-------------	-------------	-------------	-------------
14.	Cuneiform bulliform	55.61±9.70	31.23±4.43	1635.51±453.77	152.52±22.54	1.71±0.07	-------------	-------------	-------------	-------------	-------------	50.42±3.33	29.86±3.42	1300.50±201.58	147.82±9.78	1.56±0.13	34.41±2.87	25.37±1.64	777.80±101.81	113.65±7.57	1.31±0.07
15.	Cylindrical	69.80±10.27	19.87±2.14	1372.83±231.06	172.88±19.95	3.46±0.44	-------------	-------------	-------------	-------------	-------------	-------------	-------------	-------------	-------------	-------------	-------------	-------------	-------------	-------------	-------------
16.	Echinate elongate	-------------	-------------	-------------	-------------	-------------	-------------	-------------	-------------	-------------	-------------	-------------	-------------	-------------	-------------	-------------	59.20±6.76	7.00±0.55	456.27±59.28	167.06±12.60	8.10±0.77
17.	Elongate irregular	-------------	-------------	-------------	-------------	-------------	104.25±7.52	14.63±2.22	1746.56±234.41	232.70±16.06	4.53±1.11	108.25±8.57	17.73±2.34	1946.76±254.50	252.90±18.07	6.57±1.21	-------------	-------------	-------------	-------------	-------------
18.	Elongate with concave ends	106.21±15.72	25.47±0.71	2585.67±348.07	277.14±33.54	4.07±0.68	-------------	-------------	-------------	-------------	-------------	-------------	-------------	-------------	-------------	-------------	-------------	-------------	-------------	-------------	-------------
19.	Epidermal element with columellate extensions	-------------	-------------	-------------	-------------	-------------	-------------	-------------	-------------	-------------	-------------	-------------	-------------	-------------	-------------	-------------	106.73±12.37	64.04±9.81	6683.84±1851.77	324.26±43.27	1.64±0.21
20.	Globular echinate	23.44±2.61	20.90±2.55	537.16±138.72	85.32±10.82	1.27±0.04	-------------	-------------	-------------	-------------	-------------	-------------	-------------	-------------	-------------	-------------	-------------	-------------	-------------	-------------	-------------
21.	Globular granulate	-------------	-------------	-------------	-------------	-------------	-------------	-------------	-------------	-------------	-------------	28.02±2.24	25.01±2.70	660.68±107.14	81.93±8.77	1.21±0.04	-------------	-------------	-------------	-------------	-------------
22.	Globular polyhedral	41.09±1.52	35.64±1.31	1257.91±79.84	136.17±4.87	1.16±0.03	-------------	-------------	-------------	-------------	-------------	24.81±2.46	19.57±1.87	501.83±107.14	81.93±8.06	1.31±0.03	25.40±1.88	22.36±1.75	497.21±68.17	83.38±5.69	1.18±0.03
23.	Globular psilate	42.70±3.90	38.19±3.08	1415.16±213.24	132.46±9.31	1.22±0.05	26.85±2.19	19.72±1.45	496.82±64.18	78.53±5.03	1.32±0.08	-------------	-------------	-------------	-------------	-------------	-------------	-------------	-------------	-------------	-------------
24.	Half moon	-------------	-------------	-------------	-------------	-------------	64.15±3.40	30.70±1.92	1448.68±41.71	162.94±4.35	1.91±0.21	-------------	-------------	-------------	-------------	-------------	-------------	-------------	-------------	-------------	-------------
25.	Horned tower	-------------	-------------	-------------	-------------	-------------	-------------	-------------	-------------	-------------	-------------	19.50±1.76	11.71±0.83	220.34±28.33	59.60±4.29	1.71±0.12	-------------	-------------	-------------	-------------	-------------
26.	Nodular bilobate	-------------	-------------	-------------	-------------	-------------	-------------	-------------	-------------	-------------	-------------	25.45±0.95	6.46±0.60	136.40±17.19	66.24±2.05	4.56±0.33	-------------	-------------	-------------	-------------	-------------
27.	Ovate	-------------	-------------	-------------	-------------	-------------	40.15±2.00	21.00±1.28	766.01±65.00	115.30±4.91	1.97±0.14	-------------	-------------	-------------	-------------	-------------	-------------	-------------	-------------	-------------	-------------
28.	Parallelepipedal bulliform cell	52.62±2.10	28.58±1.58	1355±94.62	151.88±6.31	1.69±0.11	-------------	-------------	-------------	-------------	-------------	-------------	-------------	-------------	-------------	-------------	-------------	-------------	-------------	-------------	-------------
29.	Polylobate irregular	-------------	-------------	-------------	-------------	-------------	-------------	-------------	-------------	-------------	-------------	-------------	-------------	-------------	-------------	-------------	28.04±1.42	5.84±0.32	149.95±19.63	67.29±6.25	4.10±0.38
30.	Prickle hair	-------------	-------------	-------------	-------------	-------------	-------------	-------------	-------------	-------------	-------------	43.06±12.04	4.53±1.04	202.11±34.05	111.43±11.02	4.90±0.90	-------------	-------------	-------------	-------------	-------------
31.	Rectangular	54.50±6.02	36.30±3.93	1844.83±377.90	163.00±15.41	1.40±0.08	-------------	-------------	-------------	-------------	-------------	36.12±3.88	21.90±2.31	773.40±169.04	109.12±10.65	1.70±0.15	36.61±3.89	21.37±3.07	888.44±170.61	119.70±11.80	1.76±0.40
32.	Rondel	-------------	-------------	-------------	-------------	-------------	-------------	-------------	-------------	-------------	-------------	-------------	-------------	-------------	-------------	-------------	10.75±0.64	6.37±0.33	69.11±5.22	38.1±1.93	1.62±0.13
33.	Scutiform	-------------	-------------	-------------	-------------	-------------	44.38±4.00	23.85±2.00	88.70±98.70	128.10±9.18	1.59±0.09	45.29±2.66	26.12±2.36	954.70±108.34	129.02±6.69	1.61±0.10	48.95±4.74	20.42±1.70	736.06±66.72	130.12±9.15	2.10±0.23
34.	Sinuate elongate	-------------	-------------	-------------	-------------	-------------	95.60±5.07	11.34±0.24	1143.80±75.61	214.34±13.39	6.50±0.39	-------------	-------------	-------------	-------------	-------------	-------------	-------------	-------------	-------------	-------------
35.	Smooth elongate	66.07±7.25	8.21±0.60	566.58±91.41	148.44±15.08	7.45±0.70	165.35±35.30	26.26±4.67	4813.86±1591	390.52±81.82	7.15±1.24	-------------	-------------	-------------	-------------	-------------	70.06±10.66	7.95±0.61	554.90±117.16	159.83±23.45	8.15±0.88
36.	Tabular simple	68.18±5.00	51.16±4.03	3062.47±403.32	214.38±15.73	1.33±0.33	-------------	-------------	-------------	-------------	-------------	-------------	-------------	-------------	-------------	-------------	-------------	-------------	-------------	-------------	-------------
37.	Tabular irregular	-------------	-------------	-------------	-------------	-------------	-------------	-------------	-------------	-------------	-------------	83.12±5.08	53.02±7.20	3510.09±587.87	239.77±16.50	1.82±0.20	-------------	-------------	-------------	-------------	-------------
38.	Trapezoid	58.76±3.43	37.63±1.88	1830.38±134.19	173.02±6.84	1.50±0.10	50.00±2.44	31.74±1.47	1403.81±145.07	153.29±9.63	1.48±0.07	47.40±5.85	30.16±2.66	1270.812±215.8	145.19±14.94	1152±0.07	45.65±5.15	33.83±3.58	1469.20±266.48	153.05±13.29	1.47±0.14
39.	Triangular	59.01±8.92	32.68±3.17	1576.23±362.25	165.70±21.25	1.56±0.10	-------------	-------------	-------------	-------------	-------------	-------------	-------------	-------------	-------------	-------------	-------------	-------------	-------------	-------------	-------------

The entries in the Table indicate Mean±SE & [-------]=Absence of phytolith morphotype; A/R=Aspect ratio & Prem=Perimeter

Supplementary Table 5C: Morphometric measurements of phytolith morphotypes of *Setaria viridis* (L.) P. Beauv.

S. No	Phytolith Morphotypes	*Setaria viridis* (L.) P. Beauv.																			
		ROOT					CULM					LEAF					SYNFLORESCENCE				
		Length	Width	Area	Prem	A/R	Length	Width	Area	Prem	A/R	Length	Width	Area	Prem	A/R	Length	Width	Area	Prem	A/R
1.	Acicular	------------	------------	------------	------------	------------	------------	------------	------------	------------	------------	------------	------------	------------	------------	------------	56.11±5.90	6.017±0.62	366.74±65.70	123.94±12.85	6.66±0.68
2.	Bilobate class II	------------	------------	------------	------------	------------	------------	------------	------------	------------	------------	15.33±1.10	5.55±0.36	74.14±7.40	47.32±2.31	2.45±0.65	22.07±1.26	14.03±1.08	268.96±23.75	77.91±3.40	1.68±0.11
3.	Bilobate class IV	------------	------------	------------	------------	------------	------------	------------	------------	------------	------------	17.60±0.65	6.67±0.47	113.87±6.54	58.87±1.87	3.08±0.07	------------	------------	------------	------------	------------
4.	Bilobate class V	------------	------------	------------	------------	------------	------------	------------	------------	------------	------------	10.65±0.38	5.90±0.23	66.20±4.41	41.90±0.93	2.15±0.18	------------	------------	------------	------------	------------
5.	Bilobate class VI	------------	------------	------------	------------	------------	------------	------------	------------	------------	------------	13.28±0.49	9.21±0.34	121.40±3.43	56.14±3.12	1.21±0.65	------------	------------	------------	------------	------------
6.	Bilobate class VII	------------	------------	------------	------------	------------	------------	------------	------------	------------	------------	14.84±0.165	8.63±0.29	110.94±4.30	49.93±1.32	1.68±0.09	------------	------------	------------	------------	------------
7.	Bilobate class VIII	------------	------------	------------		------------	------------	------------	------------	------------	------------	13.60±0.30	5.41±0.24	58.84±2.52	37.23±1.12	2.05±0.07	------------	------------	------------	------------	------------
8.	Blocky irregular	50.84±2.61	30.21±2.6	1374.4±163.16	146.07±7.55	1.51±0.05	48.37±5.04	30.72±2.72	1459.46±350.13	150±17.71	1.47±0.08	58.0±10.70	37.91±4.38	2558.75	182.55±32.55	1.46±0.07	61.70±5.84	33.13±1.63	1883.73±242.52	177.57±13.78	1.70±0.11
9.	Blocky polyhedral	73.93±15.23	48.10±11	4312.45±2215.20	219.48±41.83	1.44±0.06	47.29±3.77	31.94±2.88	1254.90±158.18	139.74±9.03	1.50±0.70	44.08±2.66	30.87±1.24	1249.10±109.27	139.70±6.72	1.50±0.08	------------	------------	------------	------------	------------
10.	Carinate	------------	------------	------------	------------	------------	57.12±2.53	17.22±1.10	980.13±141.06	145.40±11.90	2.85±0.25	------------	------------	------------	------------	------------	41.66±3.12	10.94±1.51	486.53±81.07	99.92±7.82	3.19±0.25
11.	Clavate	------------	------------	------------	------------	------------	43.51±2.53	14.36±1.00	584.18±65.65	114.22±5.71	2.91±0.16	45.72±7.83	12.43±1.12	620.60±155.88	114.56±17.68	3.16±0.46	------------	------------	------------	------------	------------
12.	Cuboid	42.16±3.08	26±2.19	1053±134.32	129.13±7.64	1.68±0.16	34.45±3.98	20.77±1.36	827.42±140.90	112.32±9.25	1.58±0.10	39.93±3.45	32.16±1.94	1268.11±136.53	140.09±8.66	1.24±0.05	------------	------------	------------	------------	------------
13.	Cuneiform bulliform	------------	------------	------------	------------	------------	48.68±4.91	28.94±2.26	1204.37±220.10	139.81±12.60	1.61±0.07	------------	------------	------------	------------	------------	44.50±1.22	25.63±1.88	968.70±92.32	129.65±5.62	1.68±0.08
14.	Cylindrical	------------	------------	------------	------------	------------	------------	------------	------------	------------	------------	------------	------------	------------	------------	------------	61.56±5.73	9.36±0.77	642.07±113.47	140.70±11.60	5.73±0.37
15.	Echinate elongate	------------	------------	------------	------------	------------	------------	------------	------------	------------	------------	54.21±4.48	11.63±1.25	627.47±93.65	133.20±11.21	4.99±0.62	------------	------------	------------	------------	------------
16.	Elongate irregular	------------	------------	------------	------------	------------	56.81±4.23	16.98±1.25	1014.87±121.28	154.83±9.72	3.10±0.28	------------	------------	------------	------------	------------	------------	------------	------------	------------	------------
17.	Epidermal element	------------	------------	------------	------------	------------	------------	------------	------------	------------	------------	------------	------------	------------	------------	------------	88.48±10.76	45.46±2.67	3839.11±464.74	265.96±21.04	1.85±0.27
18.	Epidermal element with short silica cells and stomata	------------	------------	------------	------------	------------	------------	------------	------------	------------	------------	------------	------------	------------	------------	------------	87.85±8.00	51.09±3.05	3970.24±338.31	270.00±12.94	1.64±0.15
19.	Epidermal element with undulated ridges	------------	------------	------------	------------	------------	65.17±2.63	28.80±3.09	1876.38±193.62	194.70±8.81	2.31±0.20	------------	------------	------------	------------	------------	------------	------------	------------	------------	------------
20.	Epidermal papillate	------------	------------	------------	------------	------------	------------	------------	------------	------------	------------	------------	------------	------------	------------	------------	77.24±7.98	49.02±4.22	3760.74±727.82	241.50±23.36	1.44±0.07
21.	Globular echinate	------------	------------	------------	------------	------------	18.50±1.83	16.96±1.32	324.08±52.32	69.65±5.41	1.20±0.03	32.51±2.05	27.91±1.90	927.52±136.97	113.41±8.57	1.22±0.03	------------	------------	------------	------------	------------
22.	Globular granulate	39.80±3.84	29.63±3.0	1202.91±258.15	125.91±13.82	1.27±0.03	------------	------------	------------	------------	------------	30.14±2.53	29.13±1.97	824.12±86.23	98.41±5.13	1.04±0.06	------------	------------	------------	------------	------------
23.	Globular polyhedral	------------	------------	------------	------------	------------	27.87±1.97	25.04±2.40	827.80±100.17	109.15±6.71	1.20±0.05	37.57±3.46	30.70±2.54	1241.07±252	129.26±12.74	1.28±0.03	34.31±2.82	31.14±1.95	1168.31±180.69	128.35±9.16	1.26±0.06
24.	Globular psilate	28.91±1.60	24.26±1.1	665.01±55.72	91.08±3.81	1.19±0.02	23.34±2.36	23.34±1.61	595.96±100.44	84.30±6.75	1.13±0.02	------------	------------	------------	------------	------------	26.31±1.82	24.48±1.00	689.352±63.63	92.47±4.3	1.11±0.03
25.	Macrohairs	------------	------------	------------	------------	------------	------------	------------	------------	------------	------------	73.06±22.04	5.53±1.04	402.11±34.05	171.43±11.02	5.90±0.90	------------	------------	------------	------------	------------
26.	Nodular bilobate	------------	------------	------------	------------	------------	------------	------------	------------	------------	------------	22.04±1.10	6.93±0.90	131.22±9.15	61.06±3.05	3.45±0.03	------------	------------	------------	------------	------------
27.	Oblong	48.81±3.50	21.60±1.53	952.55±115.82	130.02±9.42	2.09±0.03	------------	------------	------------	------------	------------	------------	------------	------------	------------	------------	43.37±3.04	19.96±2.25	795.60±121.20	111.23±7.75	2.06±0.07
28.	Ovate						------------	------------	------------	------------	------------	44.21±5.73	19.13±1.20	760.09±144.90	114.75±12.03	2.27±0.14	------------	------------	------------	------------	------------
29.	Parallelepipedal bulliform cell	37.07±3.00	19.24±1.90	708.0±101.70	109.09±7.67	1.95±0.17	------------	------------	------------	------------	------------	------------	------------	------------	------------	------------	31.35±3.68	16.67±1.65	509.33±75.19	90.90±8.04	1.93±0.16
30.	Plates	------------	------------	------------	------------	------------	25.58±2.20	11.37±0.60	311.41±44.14	75.02±5.45	2.10±0.16	24.90±1.47	9.70±0.61	222.40±13.84	70.30±3.13	2.76±0.29	------------	------------	------------	------------	------------
31.	Prickle hair	------------	------------	------------	------------	------------	------------	------------	------------	------------	------------	40.16±5.12	12.84±1.29	484.41±116.48	98.78±12.44	2.78±0.24	------------	------------	------------	------------	------------
32.	Prickly elongate	------------	------------	------------	------------	------------	------------	------------	------------	------------	------------	------------	------------	------------	------------	------------	74.60±5.63	11.82±1.18	680.57±68.60	236.70±16.53	8.04±0.86
33.	Rectangular	40.0±2.72	19.53±2.25	815.75±137.36	115.77±8.67	2.08±0.19	42.76±2.84	15.90±1.60	748.21±97.27	121.23±7.30	2.73±0.35	------------	------------	------------	------------	------------	------------	------------	------------	------------	------------
34.	Rondel	------------	------------	------------	------------	------------	18.96±1.64	12.77±0.96	246.80±31.26	65.40±4.37	1.56±0.08	------------	------------	------------	------------	------------	------------	------------	------------	------------	------------
35.	Scutiform	43.83±2.40	14.87±0.83	646.52±86.86	113.85±6.98	2.66±0.20	------------	------------	------------	------------	------------	39.90±3.67	15.91±1.38	559.99±76.94	107.23±8.51	2.16±0.15	41.71±2.20	18.32±0.90	672.81±56.88	122.25±3.50	2.34±0.17
36.	Sinuate elongate	------------	------------	------------	------------	------------	------------	------------	------------	------------	------------	54.94±3.16	9.44±0.47	535.24±62.78	136.26±7.29	5.63±0.43	------------	------------	------------	------------	------------
37.	Smooth elongate	------------	------------	------------	------------	------------	59.25±5.13	7.60±0.92	463.50±55.40	134.89±10.15	7.84±1.26	83.46±10.82	170.25±160.93	835.81±190.78	163.05±27.90	6.66±1.07	61.64±7.84	7.84±0.72	509.96±102.61	144.57±17.03	8.01±0.78
38.	Tabular irregular	------------	------------	------------	------------	------------	------------	------------	------------	------------	------------	41.65±2.20	28.63±1.50	1064.02±131.62	126.77±8.05	1.35±0.07	------------	------------	------------	------------	------------
39.	Tabular polyhedral	------------	------------	------------	------------	------------	51.33±3.82	34.40±2.27	1680.80±207.54	158.41±9.45	1.42±0.10	49.70±4.71	32.97±2.58	1477.70±266.77	149.47±13.02	1.35±0.05	------------	------------	------------	------------	------------
40.	Trapezoid	49.42±7.58	31.09±3.78	1538.77±570.81	150.5±23.31	1.52±0.10	42.26±6.05	26.54±2.02	1226.92±231.53	136.16±13.27	1.42±0.06	41.77±3.32	27.24±2.22	1049.01±140.57	123.63±9.91	1.45±0.09	47.97±4.12	32.70±4.11	1374.52±239.60	140.52±10.87	1.50±0.08
41.	Triangular	46.11±4.76	23.66±1.71	905.01±176.25	126.90±11.70	1.90±0.15	25.66±1.80	19.56±2.03	446.58±48.10	93.73±5.97	1.50±0.09	------------	------------	------------	------------	------------	26.17±2.14	17.64±1.28	312.46±23.74	75.95±2.92	1.50±0.11

The entries in the Table indicate Mean±SE & [-------]=Absence of phytolith morphotype; A/R=Aspect ratio & Prem=Perimeter

Supplementary **Table 6. Elemental composition of phytolith in various parts of Setaria spp.**

Species	*Setaria pumila* (Poir.) Roem. & Schult.								*Setaria verticillata* (L.) P. Beauv.								*Setaria viridis* (L.) P. Beauv.							
**Plant Part**	**Root**		**Culm**		**Leaf**		**Inflorescence**		**Root**		**Culm**		**Leaf**		**Inflorescence**		**Root**		**Culm**		**Leaf**		**Inflorescence**	
**Elements**	**WT%**	**AT%**	**WT%**	**AT%**	**WT%**	**AT%**	**WT%**	**AT%**	**WT%**	**AT%**	**WT%**	**AT%**	**WT%**	**AT%**	**WT%**	**AT%**	**WT%**	**AT%**	**WT%**	**AT%**	**WT%**	**AT%**	**WT%**	**AT%**
Aluminum (Al)	3.77±2.3	2.92±1.79	----------	----------	0.59±0.59	0.39±0.39	2.36±2.36	2.07±2.07	4.62±1.84	3.19±1.28	----------	----------	3.43±3.12	2.47±2.27	----------	----------	4.84±3.49	3.85±2.99	0.63±0.63	0.51±0.51	7.32±3.09	5.86±2.50	1.99±1.99	1.62±1.62
Barium (Ba)	----------	----------	----------	----------	----------	----------	----------	----------	0.16±0.16	0.02±0.02	----------	----------	----------	----------	----------	----------	----------	----------	----------	----------	----------	----------	----------	----------
Carbon (C)	----------	----------	8.17±8.17	10.2±10.2	4.23±4.23	6.31±6.31	17.09±10.9	20.63±13.1	30.36±4.99	42.46±4.72	55.96±7.32	66.93±5.99	35.92±6.24	49.24±6.26	34.75±4.28	46.82±4.31	6.86±6.86	9.33±9.33	----------	----------	----------	----------	6.88±6.88	9.13±9.13
Calcium (Ca)	0.18±0.18	0.10±0.10	1.02±0.63	0.44±0.27	0.81±0.81	0.36±0.36	0.10±0.10	0.06±0.06	0.45±0.45	0.21±0.21	0.03±0.03	0.01±0.01	----------	----------	----------	----------	----------	----------	0.93±0.93	0.48±0.48	----------	----------	----------	----------
Chlorine (Cl)	0.32±0.32	0.19±0.19	1.172±0.65	0.63±0.36	2.00±0.58	1.22±0.37	0.34±0,24	0.22±0.16	0.21±0.13	0.09±0.06	0.62±0.06	0.26±0.36	0.16±0.10	0.07±0.04	1.0±0.13	0.46±0.064	----------	----------	----------	----------	----------	----------	----------	----------
Copper (Cu)	1.30±0.13	0.43±0.05	2.88±0.92	0.92±0.30	0.98±0.98	0.31±0.31	----------	----------	0.53±0.06	0.15±0.02	0.66±0.08	0.16±0.02	0.68±0.11	0.19±0.04	0.71±0.06	0.18±0.02	3.54±1.44	1.18±0.48	3.85±0.65	1.29±0.22	----------	----------	2.73±0.24	0.88±0.10
Iron (Fe)	0.24±0.24	0.09±0.09	----------	----------	1.94±0.79	0.53±0.12	0.63±0.63	0.26±0.26	0.20±0.10	0.06±0.03	----------	----------	0.67±0.42	0.22±0.14	----------	----------	0.59±0.37	0.22±0.14	----------	----------	0.58±0.36	0.23±0.14	----------	----------
Potassium (K)	0.46±0.15	0.25±0.08	----------	----------	----------	----------	1.66±0.37	0.87±0.24	5.35±2.64	3.23±1.45	----------	----------	2.69±2.32	1.33±1.17	1.08±0.48	0.48±0.22	2.49±1.68	1.31±0.97	0.62±0.40	0.34±0.22	3.92±2.02	2.19±1.14	0.73±0.45	0.40±0.25
Magnesium (Mg)	----------	----------	----------	----------	0.57±0.57	0.42±0.42	----------	----------	----------	----------	----------	----------	0.12±0.09	0.09±0.72	0.05±0.05	0.04±0.04	----------	----------	----------	----------	----------	----------	----------	----------
Sodium (Na)	2.38±1.46	2.16±1.32	----------	----------	----------	----------	----------	----------	0.45±0.33	0.45±0.32	----------	----------	----------	----------	----------	----------	----------	----------	1.06±1.06	1.21±1.21	1.26±1.26	1.13±1.13	1.12±1.12	1.06±1.06
Oxygen (O)	46.7±1.66	60.78±1.61	47.36±4.42	58.27±5.8	40.33±1.74	52.88±3.0	45.03±4.24	54.83±8.13	32.86±2.24	35.26±1.62	24.11±2.44	22.46±3.02	26.64±3.28	28.32±3.82	34.33±1.80	35.27±2.01	41.51±7.07	53.70±4.90	48.52±2.59	64.55±3.71	43.38±1.91	57.66±1.74	41.78±1.46	53.36±3.34
Phosphorus (P)	----------	----------	----------	----------	----------	----------	----------	----------	----------	----------	----------	----------	----------	----------	0.08±0.08	0.05±0.05	----------	----------	----------	----------	----------	----------	----------	----------
Rubidium (Rb)	----------	----------	----------	----------	----------	----------	----------	----------	----------	----------	----------	----------	----------	----------	----------	----------	----------	----------	3.54±3.54	0.91±0.91	----------	----------	----------	----------
Sulphur (S)	----------	----------	----------	----------	----------	----------	----------	----------	0.14±0.11	0.07±0.05	----------	----------	----------	----------	0.064±0.064	0.032±0.032	----------	----------	----------	----------	----------	----------	----------	----------
Silicon (Si)	44.6±2.75	33.09±2.12	39.40±10.1	29.58±8.25	49.53±5.86	37.58±5.0	34.38±6.57	24.67±5.58	24.67±2.85	15.54±2.28	18.63±5.06	10.18±3.05	29.60±3.0	18.01±2.17	27.93±3.98	16.68±2.96	40.02±7.07	30.31±5.86	40.97±5.63	30.31±5.86	43.33±4.97	32.84±3.74	44.78±6.11	33.55±5.51
Titanium (Ti)	----------	----------	----------	----------	----------	----------	0.30±0.30	0.15±0.15	----------	----------	----------	----------	----------	----------	----------	----------	0.14±0.14	0.06±0.06	----------	----------	0.20±0.20	0.09±0.09	----------	----------

WT%= Weight percentage; AT%=Atomic percentage; The entries in the table indicate mean± Standard error

Supplementary Table 7: Elemental composition of soils samples from the collection sites of three congeneric species of *Setaria* P. Beauv.

S.No.	Elements	Soil samples from collection sites for the species					
		Setaria pumila		*Setaria verticillata*		*Setaria viridis*	
		WT%	AT%	WT%	AT%	WT%	AT%
1.	**Aluminium**	5.97±1.14	4.37±0.94	5.27±1.16	3.78±0.96	13.44±9.09	13.05±10.37
3.	**Calcium**	2.67±0.90	1.31±0.45	1.98±0.27	0.94±0.17	0.99±0.56	0.49±0.32
2.	**Carbon**	10.55±10.55	12.48±12.48	34.19±10.52	44.14±13.29	13.55±8.79	19.64±12.42
4.	**Copper**	-----------	-----------	-----------	-----------	2.26±1.35	0.85±0.50
5.	**Iron**	3.83±1.52	1.66±0.47	4.23±1.26	1.48±0.50	3.59±1.55	1.49±0.87
7.	**Magnesium**	1.48±0.31	1.22±0.28	1.10±0.40	0.90±0.34	0.55±0.37	0.20±0.20
9.	**Oxygen**	54.05±4.34	65.06±8.06	32.44±13.38	35.14±15.03	42.16±9.20	44.97±11.07
10.	**Phosphorus**	-----------	-----------	-----------	-----------	0.04±0.04	0.02±0.02
6.	**Potassium**	2.56±0.55	1.30±0.30	1.98±0.49	0.98±0.27	4.14±1.90	2.44±1.17
12.	**Silicon**	17.51±3.20	12.30±2.50	18.54±2.34	12.51±2.10	18.89±8.44	16.58±9.98
8.	**Sodium**	0.20±0.20	0.172±0.172	0.072±0.072	0.48±0.48	-----------	-----------
11.	**Sulphur**	-----------	-----------	-----------	-----------	0.28±0.19	0.19±0.13
13.	**Titanium**	0.31±0.13	0.98±0.87	0.204±0.129	0.09±0.05	0.082±0.082	0.04±0.04
14.	**Zinc**	0.012±0.012	0.002±0.002	-----------	-----------	-----------	-----------

Supplementary Table 8: X-Ray Diffraction patterns of phytoliths from different parts of *Setaria* spp

**Silica Minerals**	**Codes**	***Setaria pumila***				***Setaria verticillata***				***Setaria viridis***			
		ROOT	CULM	LEAF	SYNFLO	ROOT	CULM	LEAF	SYNFLO	ROOT	CULM	LEAF	SYNFLO
Zeolite	A	+	+	--	--	+	--	+	--	--	--	--	--
Tridymite	B	+	+	+	+	+	--	+	--	+	+	+	+
Quartz	C	+	+	+	+	+	+	--	--	+	+	+	+
Cristobalite	D	+	+	+	--	+	--	--	--	--	--	+	+
Stishovite	E	+	--	+	--	--	--	--	--	--	--	--	--
Coesite	F	+	--	+	--	+	--	+	--	+	--	--	+
SiO2	G	+	+	+	+	+	+	--	+	+	+	+	+
Ferririte	H	--	--	--	--	--	--	--	--	+	--	--	--

Supplementary Table 9: FTIR peak positions showing different functional groups in phytoliths of *Setaria* spp.

S.NO.	PEAK POSITIONS (Cm^{-1})	TYPE OF VIBRATION	STRUCTURAL UNIT	*Setaria pumila*	*Setaria verticillata*	*Setaria viridis*
1.	445.67−472.00	**δO—Si---O**	--O--Si—O--	+	+	+
2.	530.39−563.18	**νSi—O**	**SiO_2 defects**	------	+	------
3.	637.48−699.54	ν_sSi—O--Si	≡Si—O—Si≡	+	+	+
4.	712.70−801.08	ν_sSi—O	≡Si—O—Si≡	+	+	+
5.	827.41−851.86	**νSi—C**	Si—R	+	------	+
6.	906.48	ν_βSi—O	Free Si—O^-	------	+	------
7.	979.73	ν_βSi—O	≡Si—OH	+	------	+
8..	1080.06−1094.44	**ν_{as}Si—O—Si (LO mode)**	≡Si—O—Si≡	+	+	+
9..	1164.92	**ν_{as}Si—O—Si(TO mode)**	≡Si—O—Si≡	------	+	------
10.	1218.93	**δ_sC---H**	Si---R	------	+	-----
11.	1323.08−1332.72	**δ—CH_2---**	R	------	+	------
12..	1463.02	**δ_{as}—C—H, δ_sC---H**	--CH_3—CH_2	------	------	+
13.	1513.79−1538.24	**ν_βC=C**	Si--ph	+	------	+
14.	1602.17−1616.24	**ν_βC=C**	Si---CH—CH_2	+	+	+
15.	1628.50−1641.66	**δH---O---H**	H---O---H	+	+	+
16.	1701.84	**νβSi---C**	Si--R	-----	-----	+
17.	1743.21−1933.14		***Al_2O_3.SiO_2***	+	-----	+
18.	2339.32−2366.49	**ν_βSi---C**	Si--R	+	+	+
19.	2825.52	**ν_sC---H**	---CH_2	------	+	-----
20.	2952.74−2987.53	**ν_{as}C---H**	---C H_2	+	+	-----
21.	3006.82−3271.05	**ν**O---H	H—O—H…H_2O	------	+	-----
22.	3346.27−3597.36	O----H & Si----OH	H—O—H…H_2O and ≡SiO--- H….H_2O	+	+	+
23.	3661.30−3941.50	Si----OH	≡SiO---H….H_2O	+	------	+

ν= stretching vibration; νs=symmetric stretching vibration; ν_{as}=antisymmetric stretching vibration; δ=deformation vibration; δ_s=symmetric deformation vibration (bending); δ_{as}=anti deformation vibration (bending); ν_β=inplane stretching vibration; LO=longitudinal optical; TO=transversal optical.
