# Supplementary figures and images for: Taxonomic Demarcation of Setaria pumila (Poir.) Roem. & Schult., S. verticillata (L.) P. Beauv., and S. viridis (L.) P. Beauv. (Cenchrinae, Paniceae, Panicoideae, Poaceae) From Phytolith Signatures
Source: Front Plant Sci. 2018 Jun 22;9:864. doi: 10.3389/fpls.2018.00864 (PMC6024613; doi:10.3389/fpls.2018.00864)

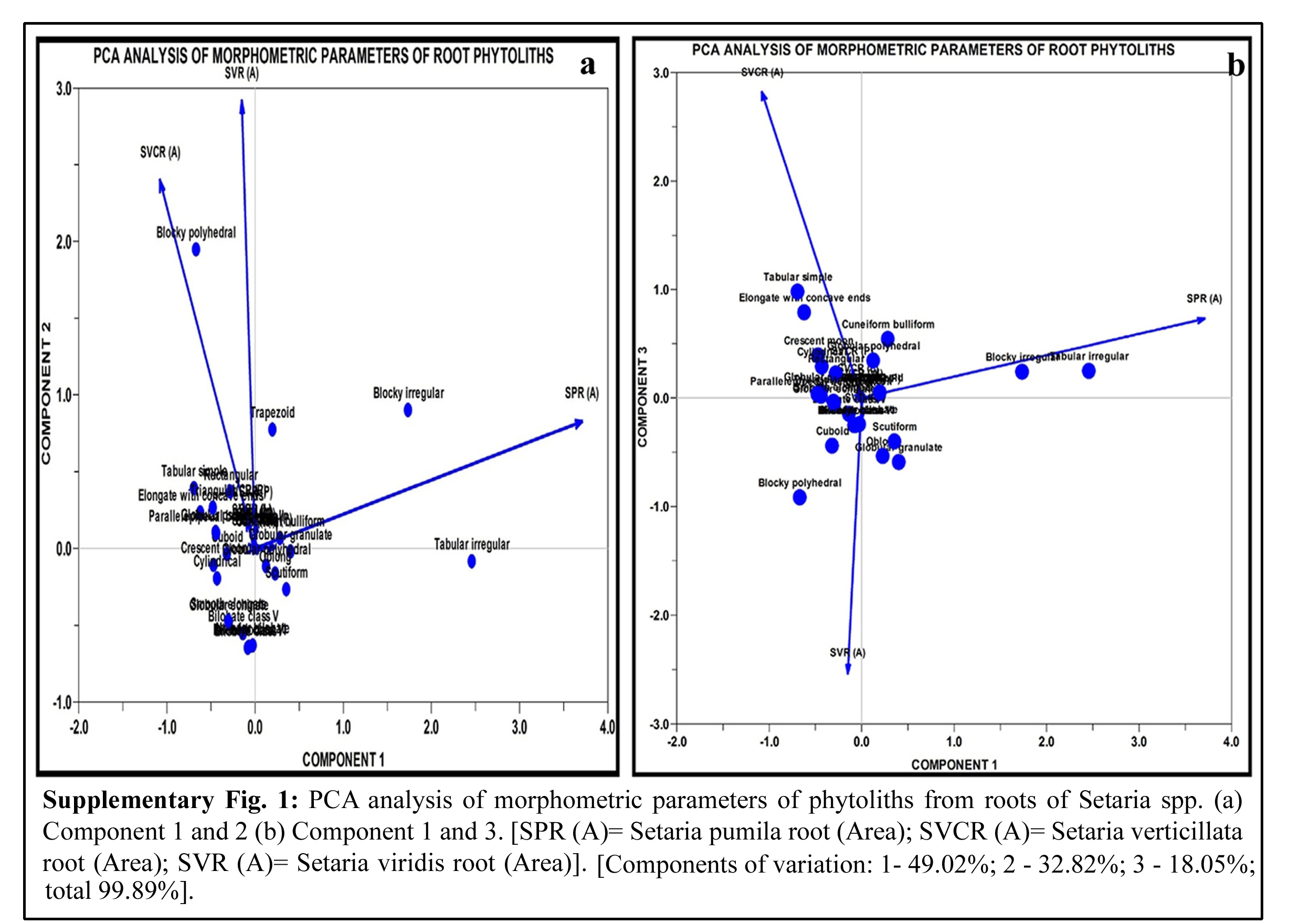

Supplement: Supplementary file 2 [file Image_1.jpg]

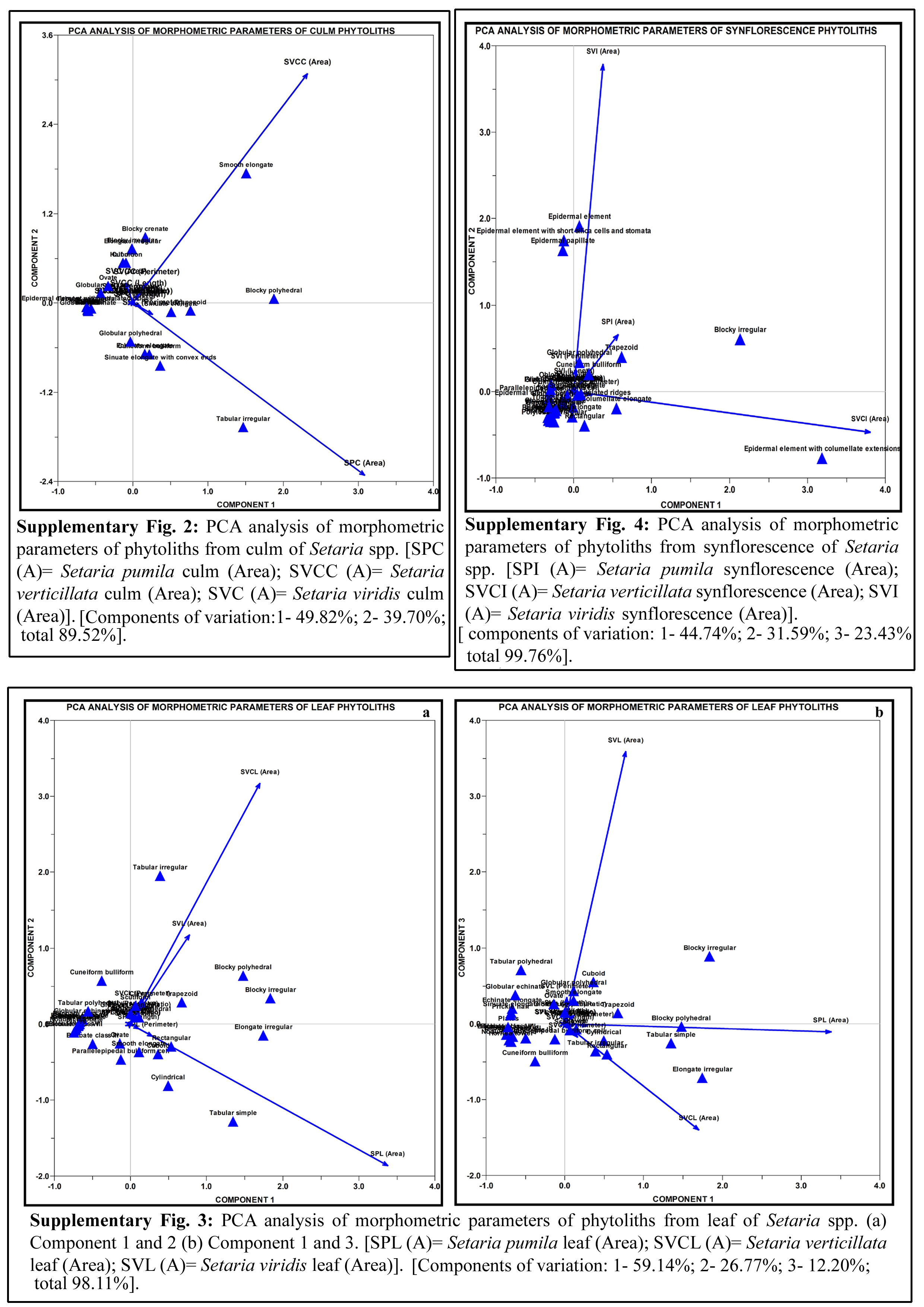

Supplement: Supplementary file 3 [file Image_2.jpg]

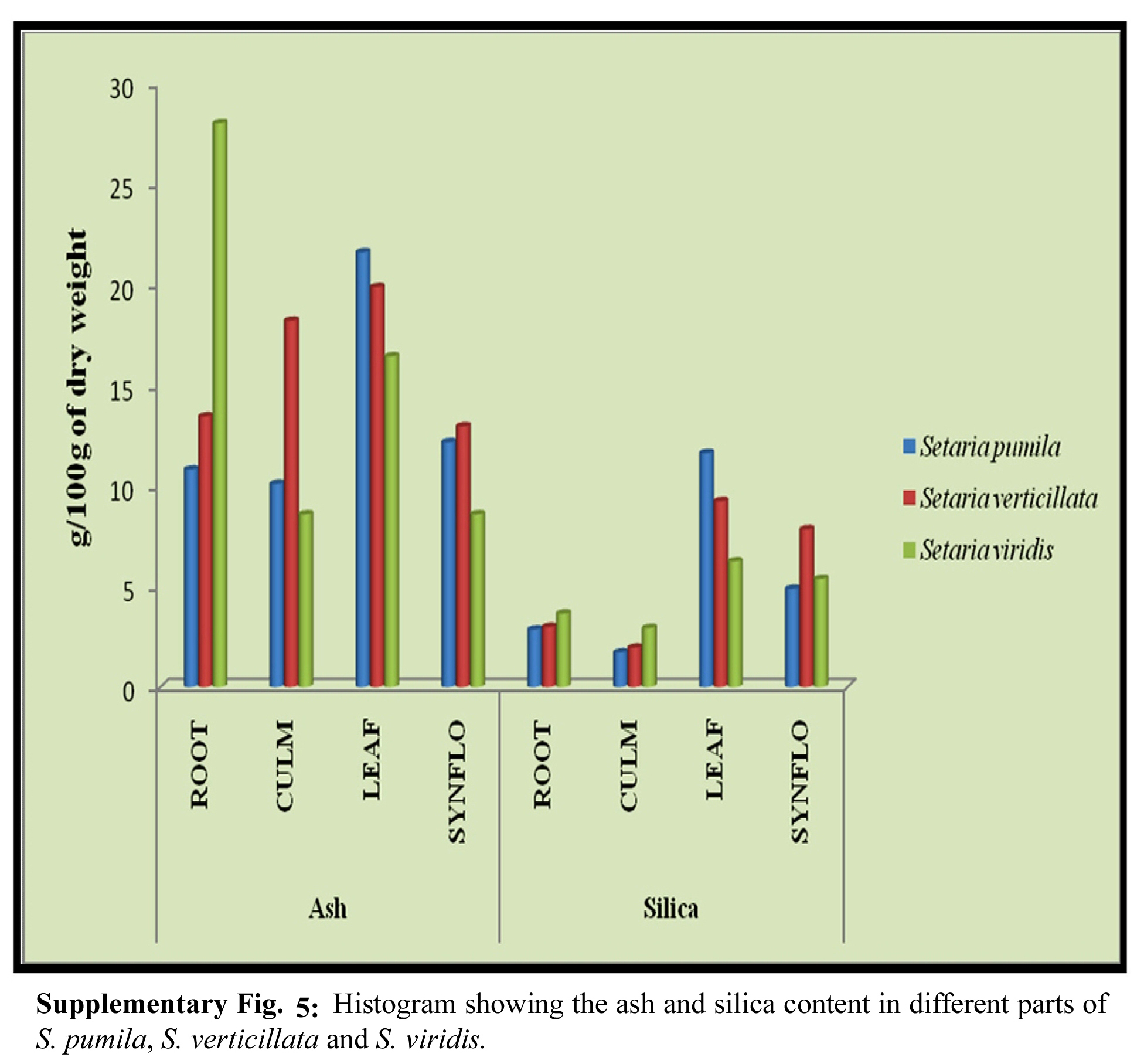

Supplement: Supplementary file 4 [file Image_3.jpg]
